# The effects of probiotic and synbiotic supplementation on inflammation, oxidative stress, and circulating adiponectin and leptin concentration in subjects with prediabetes and type 2 diabetes mellitus: a GRADE-assessed systematic review, meta-analysis, and meta-regression of randomized clinical trials

**DOI:** 10.1007/s00394-022-03012-9

**Published:** 2022-10-14

**Authors:** Kaveh Naseri, Saeede Saadati, Farahnaz Ghaemi, Damoon Ashtary-Larky, Omid Asbaghi, Amir Sadeghi, Reza Afrisham, Barbora de Courten

**Affiliations:** 1 Gastroenterology and Liver Diseases Research Center, Research Institute for Gastroenterology and Liver Diseases, Shahid Beheshti University of Medical Sciences, Tehran, Iran; 2 Department of Medicine, School of Clinical Sciences, Monash University, 246 Clayton Rd, Clayton, Melbourne, VIC 3168 Australia; 3 Department of Microbiology, Kerman Branch, Islamic Azad University, Kerman, Iran; 4 Nutrition and Metabolic Diseases Research Center, Ahvaz Jundishapur University of Medical Sciences, Ahvaz, Iran; 5 Cancer Research Center, Shahid Beheshti University of Medical Sciences, Tehran, Iran; 6 Department of Clinical Laboratory Sciences, Faculty of Allied Medicine, Tehran University of Medical Sciences, Tehran, Iran; 7 School of Health and Biomedical Sciences, RMIT University, Bundoora, Australia

**Keywords:** Probiotic, Synbiotic, T2DM, Prediabetes, Inflammation, Oxidative stress, Adipokines

## Abstract

**Purpose:**

Probiotics or synbiotics consumption have been suggested to reduce the risk of cardiovascular disease (CVD) through a decline in inflammation and oxidative stress, however, the results from studies are conflicting. This study filled this knowledge gap by evaluating randomized controlled trials (RCTs) investigating probiotics or synbiotics intake on adipokines, inflammation, and oxidative stress in patients with prediabetes and type-2 diabetes mellitus (T2DM).

**Methods:**

We systematically did search up to March 2022 in PubMed/Medline, Scopus, ISI Web of Science, and Cochrane library. A random-effect model was applied to estimate the weighted mean difference (WMD) and 95% confidence interval (95% CI) for each outcome.

**Results:**

A total of 32 RCTs were included in the meta-analysis. This intervention led to a significant decrease in levels of C-reactive protein (CRP) (WMD − 0.62 mg/l; 95% CI − 0.80, − 0.44; *p* < 0.001), tumor necrosis factor-α (TNF-α) (WMD − 0.27 pg/ml; 95% CI − 0.44, − 0.10; *p* = 0.002) and malondialdehyde (MDA) (WMD − 0.51 µmol/l; 95% CI − 0.73, − 0.30; *p* < 0.001), and also a significant increase in levels of glutathione (GSH) (WMD 69.80 µmol/l; 95% CI 33.65, 105.95; *p* < 0.001), total antioxidant capacity (TAC) (WMD 73.59 mmol/l; 95% CI 33.24, 113.95; *p* < 0.001) and nitric oxide (NO) (WMD 7.49 µmol/l; 95% CI 3.12, 11.86; *p* = 0.001), without significant alterations in interleukin-6 (IL-6) and adipokines levels.

**Conclusion:**

A consumption of probiotics or synbiotics could be a useful intervention to improve cardiometabolic outcomes through a reduced inflammation and oxidative stress in patients with prediabetes and T2DM.

**Supplementary Information:**

The online version contains supplementary material available at 10.1007/s00394-022-03012-9.

## Introduction

Type 2 diabetes mellitus (T2DM) is a global health concern with a high financial and social burden on the health care system. According to the International Diabetes Federation (IDF), T2DM now affects over 10% of the adult population, and it is projected to rise to 578 million by 2030 and 783.2 million by 2045 [1, 2]. It is reported that 5–10% of those with prediabetes develop T2DM each year [3].

It is evident that inflammation and oxidative stress are prevalent in diabetes and are key factors contributing to the progression of T2DM and diabetes complications [4]. This is partially due to enhanced intestinal permeability, which has been reported in patients with T2DM [5]. Alterations in intestinal permeability can result in higher lipopolysaccharide (LPS) concentrations in the peripheral circulation, consequently increasing inflammation and oxidative stress [4, 6, 7]. In addition, metabolic dysfunction in T2DM can result in the production of large amounts of reactive oxygen species (ROS) in mitochondria [6]. Moreover, adipokines such as adiponectin and leptin play a crucial role in regulating glucose metabolism [2]. Studies on gut microbiota demonstrated an association between gut dysbiosis (the imbalance of microbes in the gut) and several chronic diseases, including obesity, inflammatory diseases, and T2DM, and its potential role in shaping host pathophysiology responses [8–11]. Gut microbiota modulation enhances insulin and adiponectin expression and decreases low-grade inflammation in T2DM [12]. The concept of regulation of the gut microbiota with probiotics, prebiotics, and synbiotics is therefore a promising approach in the management of T2DM.

Probiotics are characterized as “living microorganisms that exert beneficial effects on the health status of host” [13]. Probiotics efficiently improve the integrity of the intestinal barrier, inhibit the release of pro-inflammatory cytokines, alter oxidative stress markers and alleviate symptoms of T2DM [14, 15]. Available data regarding the effects of probiotics supplementation on inflammatory and oxidative stress biomarkers are inconsistent with some studies indicating an inverse relation [16–18] and other not showing any relationship [15, 19, 20]. Additionally, some studies have also shown improvement in serum adipokines levels following probiotics supplementation [21], whereas other studies reported no effect [2, 3].

Synbiotics represent a combination of probiotics and prebiotics (as non-digestible food ingredients), acting synergically [22]. The beneficial effects on metabolic profiles of synbiotic administration have previously reported in patients with T2DM [23]. Despite more research into the effects of synbiotics on cardiovascular outcomes, most studies showed notable discrepancies in the current evidence. Previous evidences have proposed that the intake of synbiotics reduces inflammatory markers and oxidative stress [23–25], whereas others have found no effect on inflammatory biomarkers after the administration of synbiotics [26, 27].

We aimed to conduct the current comprehensive systematic review and meta-analysis of published randomized controlled trials (RCT)s to investigate the effects of probiotics or synbiotics consumption on inflammatory and oxidative stress biomarkers and serum adipokines concentration among patients with prediabetes and T2DM. To our knowledge, this is the first GRADE-assessed systematic review, meta-analysis, and meta-regression assessing a large number of subjects across different countries on the impact of probiotics or synbiotics supplementation on these biomarkers in patients with prediabetes and T2DM.

## Materials and methods

This systematic review was conducted and reported in accordance with the 2021 updated Preferred Reporting Items for Systematic Reviews and Meta-Analyses (PRISMA) guideline [28].

### Data sources and search strategies

A comprehensive literature search was performed independently by two investigators (K.N. and S.S.), applying the online databases including PubMed/MEDLINE, ISI Web of Science, Scopus, and Cochrane library, without specific time frames and language restriction, up to March 2022. The purpose of our search was to identify clinical trials studying the effects of probiotics or synbiotics on inflammatory, and oxidative stress biomarkers, adipokines and leptin, among patients with prediabetes or T2DM. We used the following MeSH and non-MeSH terms in our search strategy to identify potentially relevant studies: ((Probiotics OR probiotic OR Synbiotics OR synbiotic OR Lactobacillus OR Bifidobacterium) AND (Intervention OR “controlled trial” OR random OR randomized OR placebo OR randomly OR “clinical trial” OR Trial OR “randomized clinical trial” OR RCT OR trial OR trials “Cross-Over Studies” OR “Cross-Over” OR “Cross-Over Study” OR parallel OR “parallel study” OR “parallel trial”) AND (“diabetes” OR “type 2 diabetes mellitus” OR “T2DM” OR “type 2 diabetes” OR “T2D” OR “prediabetes”)) (see Supplementary Table 1 for search terms used across the various databases). Reference lists of the applicable research were manually screened to prevent any publications from being missed. Unpublished and/or non-human studies, as well as gray literature, were not included. After combining search results from different databases, duplicates were removed. EndNote X21 was used to manage the records. In addition, we conducted a manual search of studies fulfilling the eligibility criteria (i.e., searching the reference lists and citations).

### Eligibility criteria

All the eligible studies that were included in our analysis in accordance with the PICOS strategy as follows: (1) Population: individuals older than 18 years and with physician’s diagnosis of prediabetes or T2DM; (2) Intervention: consumption of probiotics and synbiotics (of any form, such as tablet, capsule, powder, honey, milk, yogurt and bread) in terms of dose and frequency; (3) Comparators: comparison with placebo, usual care, or any pharmacological or non-pharmacological intervention(s); (4) Outcome: those which reported mean changes and their standard deviations (SDs) of inflammatory biomarkers including tumor necrosis factor- α (TNF-α), c-reactive protein (CRP), interlukin-6 (IL-6), adipocytokines (adiponectin and leptin), and serum biomarkers of oxidative stress including glutathione (GSH), malondialdehyde (MDA), total antioxidant capacity (TAC), and nitric oxide (NO) over the length of the study for both probiotic or synbiotic and control groups or reported the required data for calculation of the related effect sizes; and (5) Study design: having a parallel or cross-over design in a RCT setting (Table 1). If more than one article was published for one dataset, the more complete one was included. Clinical trials with an additional intervention group were considered as two separate studies.

**Table 1:** PICOS criteria for inclusion of studies

Parameter	Inclusion criteria
Population	Individuals older than 18 years and with physician’s diagnosis of prediabetes or T2DM
Intervention	Consumption of probiotics and synbiotics (of any form, including capsule, tablet, powder, bread, milk, yogurt and honey) in terms of dose and frequency
Comparator	Comparison with placebo, usual care, or any pharmacological or non-pharmacological intervention(s)
Outcome	Those which reported mean changes and their standard deviations (SDs) of inflammatory factors including CRP, TNF-α, and IL-6, adipocytokines (adiponectin and leptin) and plasma/serum biomarkers of oxidative stress including GSH, MDA, TAC, and NO throughout the trial for both intervention and control groups or presented required information for calculation of those effect sizes
Study design	Being a RCT in either parallel or cross-over design

*T2DM* type 2 diabetes mellitus, *(SD)s* standard deviations, *CRP* c-reactive Protein, *TNF-α* tumor necrosis factor-α, *IL-6* interlukin-6, *GSH* glutathione, *MDA* malondialdehyde, *TAC* total antioxidant capacity, *NO* nitric oxide, *RCT* randomized controlled trial

The studies were unable to be considered if they: (1) had an open clinical trial design, (2) reported outcomes that were not been clearly declared, (3) designed as an experimental study, (4) had a non-experimental (case series, case studies, case–control, cross-sectional, cohort and other retrospective studies) design, and (5) were carried out on pregnant women, and children or adolescents.

### Study selection

Two researchers (K.N. and S.S.) independently assessed titles and abstracts, as well as the full-text review process for articles retrieved using the search technique, and any discrepancies about inclusion and exclusion of studies were resolved by consensus. Inclusion and exclusion criteria were developed based on a systematic process that considered the context, population, and evaluated the exposures and outcomes of the studies.

### Data extraction

A standardized, pre-piloted form (Excel) was used to extract data from the included studies. The parameters that were extracted were as follows: (a) name of the first author; (b) publication year; (c) individuals’ characteristics (mean age and sex); (d) the design of the study; (e) sample size (intervention and control groups); (f) type of probiotic and synbiotic administered; (g) dosage of probiotic and synbiotic; (h) length of intervention; (i) mean changes and their SDs of all the mentioned biomarkers throughout the trial for the intervention and control groups; (j) and the confounding variables adjusted in the analyses. If the reported units for each outcomes were less common, they were converted to the most commonly used unit. Any discrepancies and disagreements about the data extraction were determined by consensus or discussion with a third researcher (O.A.).

### Risk of bias assessment

The methodological quality of each included clinical trial was assessed using the Cochrane quality assessment tool on a domain-based evaluation in this meta-analysis [29]. This tool contained seven domains including random sequence generation, allocation concealment, reporting bias, other source of bias, blinding (participants and personnel), blinding (outcome assessment), and incomplete outcome data. Each domain was given a “high risk” rating if the study comprised methodological defects that may have an effect on its findings, a “low risk” rating if there was no defect for that domain and an “unclear risk” rating if the information was not enough to determine the impact. If the study was “low risk” in all areas, it was considered a high-quality study with an absolutely low risk of bias. The risk of bias was assessed independently by two reviewers (Supplementary Table 2). The overall fact of evidence across the studies was sorted in accordance with the GRADE guidelines (Grading of Recommendations Assessment, Development, and Evaluation) Working Group. The quality of evidence may be categorized into four classifications in accordance with the corresponding evaluation criteria: high, moderate, low, and very low [30].

### Data synthesis and analysis

In the probiotic/synbiotic and control groups, for each variable mean changes and their SDs were applied to acquire the overall related effect sizes. If no mean changes were reported, they were calculated by taking into account the changes in the concentration of each variable during the trial.

By applying the method of the previous study [31], interquartile ranges (IQRs), 95% confidence intervals (CIs), and standard errors (SEs) were converted to SDs. We also used a random-effects model that took into account variations between studies to get the overall effect sizes. *I*^2^ statistic and Cochrane’s *Q* test was applied for heterogeneity determination. *I*^2^ value > 50% or *P* < 0.05 for the *Q*-test was characterized as significant heterogeneity between studies [32, 33]. Subgroup analyses were conducted to find probable sources of heterogeneity based on the predefined variables such as intervention length (≥ 12 vs. < 12 weeks), intervention type (probiotic vs. synbiotic), participants’ health status (subjects with prediabetes vs. T2DM), baseline serum levels of CRP (≥ 3 vs. < 3 mg/l), TNF-α, IL-6, adiponectin, leptin, GSH, MDA, TAC, and NO. Also, we enforced the meta-regression to differentiate the confounders and linear relations among the effect size and sample size, duration of intervention, and intervention dosage. We used sensitivity analysis to determine whether the overall effect size depended upon a specific study. Therefore, we excluded studies one by one to determine the overall effect without that study [34]. The possibility of publication bias was investigated by the formal test of Begg, Egger regression and visual inspection of funnel plot [35]. The meta-analysis was conducted using the STATA^®^ version 14.0 (StataCorp, College Station, Lakeway, TX, USA). *p* value < 0.05 was considered a significant level.

## Results

### Study selection

**Fig. 1:**
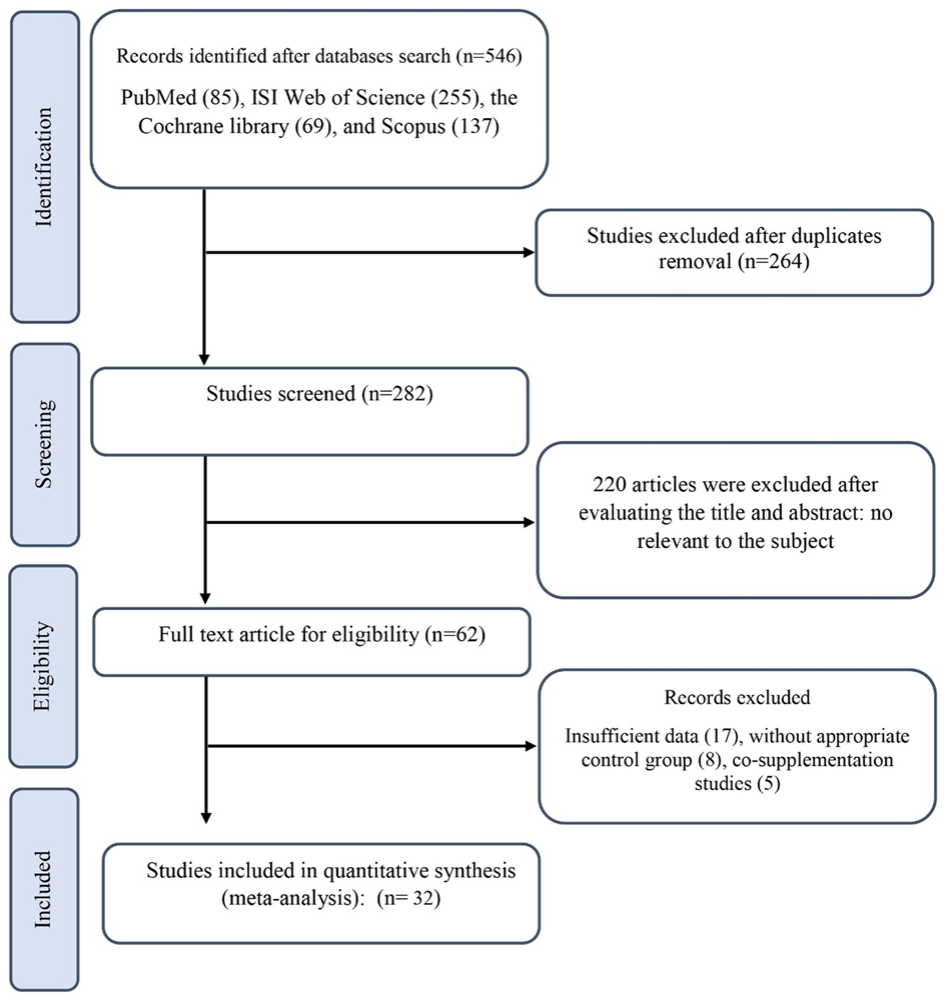
Flowchart of study selection for inclusion trials in the systematic review

The literature search and screening process performed on this systematic review is indicated in Fig. 1. A total of 546 RCTs were retrieved from the searches. Of these, 264 duplicates were removed, leaving 282 records to be screened for eligibility by title and abstract. After excluding 220 articles, 62 papers were confirmed to assess in full text. Finally, 32 studies (39 effect sizes) with 2074 subjects measuring cardiovascular outcomes were included in this review.

### Characteristics of the included studies

Table 2 is the summary of the general characteristics of the included investigations. In the current meta-analysis, 2074 participants were included (control = 956; case = 1118). Studies were published between 2012 and 2021 and were performed in Asia (n = 26) [2, 15, 20, 21, 23–27, 36–52], Europe (*n* = 3) [12, 19, 53], Africa (*n* = 1) [54], Oceania (*n* = 1) [3], and America (*n* = 1) [55]. All subjects were patients with T2DM [2, 12, 15, 19–21, 23–27, 37–55], except for two RCTs that studied individuals with prediabetes [3, 36]. Moreover, probiotic and synbiotic supplementation was used in 25 [2, 3, 12, 15, 19–21, 36–47, 49, 50, 52–55] and 7 RCTs [23–27, 48, 51], respectively. All RCTs were parallel design and their duration of supplementation ranged from 4 and 24 weeks and the sample sizes ranged from 22 to 136 participants. Also, participants’ baseline BMI ranged from 22.4 and 35.6 kg/m^2^ and ages from 46.4 to 66 years.

**Table 2:** Characteristics of the included studies

Studies	Country	Study design	Participant	Sample size and sex	Sample size		Trial duration (Week)	Means age		Means BMI		Intervention	
					**IG**	**CG**		**IG**	**CG**	**IG**	**CG**	Probiotic/synbiotic dose	Control group
Toejing et al. (2021) [2]	Thailand	paralell, R, PC, DB	T2DM	M/F (18, 18)	18	18	12	63.5 ± 5.94	61.78 ± 7.73	23.22 ± 2.72	23.05 ± 2.6	Probiotic (*L. paracasei* HII01) 50*10^9 CFU/ml	Placebo
Kanazawa et al. (2021) [26]	Japan	Paralell, R, C,	T2DM	M/F (65, 21)	44	42	24	61.1 ± 11.0	55.9 ± 10.7	29.5 ± 4.4	29.1 ± 3.7	Synbiotic mixture (10.5 g/day)	Placebo
Ismail et al. (2021) (A) [54]	Egypt	Paralell, R, PC, DB	T2DM	M/F	50	25	16	48.3 ± 12.9	46.4 ± 13.2	31.1 ± 5.3	30.2 ± 6	Probiotic (B. Animalis Dn-173 010) + Balance Diet	Yogurt
Ismail et al. (2021) (B) [54]	Egypt	Paralell, R, PC, DB	T2DM	M/F	50	25	16	48.6 ± 11.5	46.4 ± 13.2	28.5 ± 7.6	30.2 ± 6	Natural Baking Yeast (Saccharomyces Cerevisiae) + Balance Diet	Supplement
Tay et al. (2020) [3]	New Zealand	Paralell, R, PC, DB	Prediabetic	M/F (8, 18)	15	11	12	52.9 ± 8.7	54.1 ± 6.4	34.7 ± 4.9	33.6 ± 3.7	*L. rhamnosus* HNOO1 (6*109 CFU)	Placebo
Toshimitsu et al. (2020) [36]	Japan	Paralell, R, PC, DB	Prediabetic	M/F (86, 40)	62	64	12	50.6 ± 6.9	51.2 ± 7.6	24.7 ± 3.3	24.9 ± 3.2	*L. plantarum* OLI2712 (112 g (> 5*10^9^/112 g yogurt)	Yogurt
Farrokhian et al. (2019) [48]	Iran	Paralell, R, PC, DB	T2DM/Diabetes/CHD	M/F (38, 22)	30	30	12	64.2 ± 12	64 ± 11.7	32.3 ± 6	29.6 ± 4.6	Synbiotic (3 bacteria) (probiotic (2*10^9^ CFU/g/day each) + inulin)	Placebo
Mazruei Arani et al. (2019) [42]	Iran	Paralell, R, PC, DB	Diabetic nephropathy	M/F	30	30	12	62.7 ± 9.1	60.3 ± 8.5	30.3 ± 5.6	31.1 ± 4.6	Probiotic honey (2500 mg/day)	Honey
Soleimani et al. (2019) [25]	Iran	Paralell, R, PC, DB	T2DM/dialysis	M/F (42, 18)	30	30	12	62.8 ± 12.7	62.8 ± 14.8	26.4 ± 5.4	26.9 ± 4.7	Synbiotic (probiotic + inulin)	Placebo
Sabico et al. (2019) [21]	Saudi Arabia	Paralell, R, PC, DB	T2DM	M/F	31	30	24	48 ± 8.3	46.6 ± 5.9	29.4 ± 5.2	30.1 ± 5.0	Probiotic (2.5*10^9^ CFU/ml)	Placebo
Raygan et al. (2018) [38]	Iran	Paralell, R, PC, DB	T2DM/ CHD	M/F	30	30	12	60.7 ± 9.4	61.8 ± 9.8	30.3 ± 5.2	29.3 ± 4.1	Probiotic (mixture)	Placebo
Kobyliak et al. (2018) [12]	Ukraine	Paralell, R, PC, DB	T2DM	M/F	31	22	12	52.23 ± 1.74	57.18 ± 2.06	34.7 ± 1.29	35.65 ± 1.57	Probiotic (multistrain) (10 g/day)	Placebo
Mafi et al. (2018) [44]	Iran	Paralell, R, PC, DB	Diabetic nephropathy	M/F	30	30	12	58.9 ± 8.8	60.9 ± 4.4	25.3 ± 2.3	26.3 ± 3.2	Probiotic (multistrain) (8*10^9^ CFU/ml/)	Placebo
Hsieh et al. (2018) (A) [45]	Taiwan	Paralell, R, PC, DB	T2DM	M/F	25	12	12	NR	NR	NR	NR	Probiotic (*L. ruteri*. live) (2*10^9^ CFU/capsule)	Placebo
Hsieh et al. (2018) (B) [45]	Taiwan	Paralell, R, PC, DB	T2DM	M/F	25	12	12	NR	NR	NR	NR	Probiotic (*L. ruteri*. Heat-killed) (1*10^10^ CFU/capsule)	Placebo
Miraghajani et al. (2017) [41]	Iran	Paralell, R, PC,SB	T2DM/CKD	M/F (18, 22)	20	20	12	56.90 ± 1.81	53.60 ± 1.60	26.68 ± 0.71	26,58 ± 0.73	Probiotic soy milk (200 ml/day)	Soy milk
Soleimani et al. (2017) [37]	Iran	Paralell, R, PC, DB	T2DM/dialysis	M/F (40, 20)	30	30	12	54 ± 16	59.4 ± 16	25.5 ± 5.6	27 ± 6.4	Probiotic (multistrain) (mixture)	Placebo
Firouzi et al. (2017) [46]	Malaysia	Paralell, R, PC, DB	T2DM	M/F (65, 71)	68	68	12	52.9 ± 9.2	54.2 ± 8.3	29.2 ± 5.6	29.3 ± 5.3	Probiotic (multistrain) (109 3 ach/twice a day)	Sachet
Mohseni et al. (2018) [39]	Iran	Paralell, R, PC, DB	T2DM/foot ulcer	M/F (40, 20)	30	30	12	62.6 ± 9.7	58.5 ± 11	26.4 ± 3	25.3 ± 3.7	Probiotic (multistrain) (2.*10^9^ cfu/g each)	Placebo
Rezaei et al. (2017) [15]	Iran	Paralell, R, PC, DB	T2DM	M/F (44, 46)	45	45	12	50.49 ± 10.92	50.13 ± 9.2	28.9 ± 3.5	29.5 ± 1.6	Probiotic (multistrain) (300,000 (300 g/day))	Yogurt
Sato et al. (2017) [20]	Japan	Paralell, R, PC, DB	T2DM	M/F (49, 19)	34	34	12	64 ± 9.2	65 ± 8.3	24.2 ± 2.6	24.6 ± 2.6	Probiotic (multistrain) (mixture)	Placebo
Feizollahzadeh et al. (2016) [47]	Iran	Paralell, R, PC, DB	T2DM	M/F (19, 21)	20	20	12	56.9 ± 8.09	53.6 ± 7.15	26.68 ± 3.17	26.58 ± 3.26	Probiotic soy milk (*L. plantarum* A7) (200 ml/day)	Conventional soy milk
Bayat et al. (2016) (A) [50]	Iran	Paralell, R, C	T2DM	M/F (12, 28)	20	20	12	54.1 ± 9.54	46.95 ± 9.34	28.77 ± 4.59	29.75 ± 4.66	Probiotic yogurt (150 g/day)	Dietary advice
Bayat et al. (2016) (B) [50]	Iran	Paralell, R, C	T2DM	M/F (18, 22)	20	20	12	53.65 ± 6.99	51.8 ± 2.24	27.98 ± 4.2	28.95 ± 3.34	Probiotic yogurt + pumpkin (150 gr + 100 gr)	Pumpkin
Mobini et al. (2017) (A), (B) [53]	Sweden	Paralell, R, PC, DB	T2DM	M/F (17, 5)	15	7	12	64 ± 6	65 ± 5	30.6 ± 4.5	30.7 ± 4	*L. reuteri* DSM 17,938 (low dose) (1*10^8^ CFU/day)	Placebo
Mobini et al. (2017) (A), (B) [53]	Sweden	Paralell, R, PC, DB	T2DM	M/F (17, 5)	14	8	12	66 ± 6	65 ± 5	32.3 ± 3.4	30.7 ± 4	*L. reuteri* DSM 17,938 high dose) (1*10^10^ CFU/day)	Placebo
Bahmani et al. (2016) (A), (B) [51]	Iran	Paralell, R, PC, DB	T2DM	M/F (8, 33)	27	14	12	52 ± 7.2	53.4 ± 7.5	29.8 ± 5.7	30.5 ± 4.1	Probiotic (*L. sporogenes*) ((1 £ 108 CFU) 3 times a day)	Bread
Bahmani et al. (2016) (A), (B) [51]	Iran	Paralell, R, PC, DB	T2DM	M/F (7, 33)	27	13	12	51.3 ± 10.4	53.4 ± 7.5	30.8 ± 5.9	30.5 ± 4.1	Synbiotic bread (*Lactobacillus sporogenes* + inulin) ((1 £ 108 CFU + 0.07 g inulin per 1 g) 3 times a day)	Bread
Hove et al. (2015) [19]	Denmark	Paralell, R, PC, DB	T2DM	M (41)	23	18	12	58.5 ± 7.7	60.6 ± 5.2	29.2 ± 3.8	27.7 ± 3.3	Milk fermented with *L. helveticus* (Cardi04 yogurt) (300 ml/day)	Yogurt
Kooshki et al. (2015) [23]	Iran	Paralell, R, PC, DB	T2DM	M/F (16, 28)	22	22	12	53.45 ± 10.8	54.5 ± 11.10	22.79 ± 2.7	22.47 ± 2.38	Synbiotic (1 tablet day)	Placebo
Tonucci et al. (2017) [55]	Brazil	Paralell, R, PC, DB	T2DM	M/F (26, 16)	23	22	12	51.83 ± 6.64	50.95 ± 7.20	27.49 ± 3.97	27.94 ± 4.15	Probiotic (mixture) (120 g/d)	Conventional fermented goat milk
Asemi et al. (2014) [24]	Iran	Paralell, R, PC, DB	T2DM	M/F (38, 86)	62	62	6	53.1 ± 8.7	58 ± 4.1	29.6 ± 4.53	29.9 ± 5.18	Synbiotic (9 g/3 times per day)	Placebo
Mohamadshahi et al. (2014) [40]	Iran	Paralell, R, PC, DB	T2DM	M/F (10, 32)	21	21	12	53 ± 5.9	49 ± 7.08	28.36 ± 4.14	29.22 ± 3.2	Probiotic (multistrain) (300 g a day)	Yogurt
Tajadadi-Ebrahimi et al. (2014) (A) [27]	Iran	Paralell, R, PC, DB	T2DM	M/F (8, 33)	27	14	12	52 ± 7.2	53.4 ± 7.5	29.8 ± 5.7	30.5 ± 4.1	Probiotic *(L. sporogenes)* (40 g 3 times a day)	Bread
Tajadadi-Ebrahimi et al. (2014) (B) [27]	Iran	Paralell, R, PC, DB	T2DM	M/F (8, 32)	27	13	12	51.3 ± 10.4	53.4 ± 7.5	30.8 ± 5.9	30.5 ± 4.1	Synbiotic (*L. sporogenes*) + (inulin) (40 g 3 times a day + (0.07 g inulin/g))	Bread
Asemi et al. (2013) [52]	Iran	Paralell, R, PC, DB	T2DM	M/F (15, 39)	27	27	8	50.51 ± 9.82	52.59 ± 7.14	31.61 ± 6.36	30.17 ± 4.23	Probiotic (multistrain) (once per day)	Placebo
Mazloom et al. (2013) [43]	Iran	Paralell, R, PC, DB	T2DM	M/F (8, 26)	16	18	12	55.4 ± 8	51.8 ± 10.2	27.97 ± 3.81	27.24 ± 2.73	Probiotic (multistrain) (1500 mg (twice a day))	Placebo
Ejtahed et al. (2012) [49]	Iran	Paralell, R, PC, DB	T2DM	M/F (23, 37)	30	30	12	50.87 ± 7.68	51 ± 7.32	28.95 ± 3.65	29.14 ± 4.3	Probiotic (multistrain) (300 g/day)	Yogurt

*IG* intervention group, *CG* control group, *DB* double-blinded, *SB* single-blinded, *PC* placebo-controlled, *CO* controlled, *RA* randomized, *NR* not reported, *F* Female, *M* Male, *NR* not reported

### Effects of probiotic and synbiotic intake on inflammatory biomarkers

**Fig. 2:**
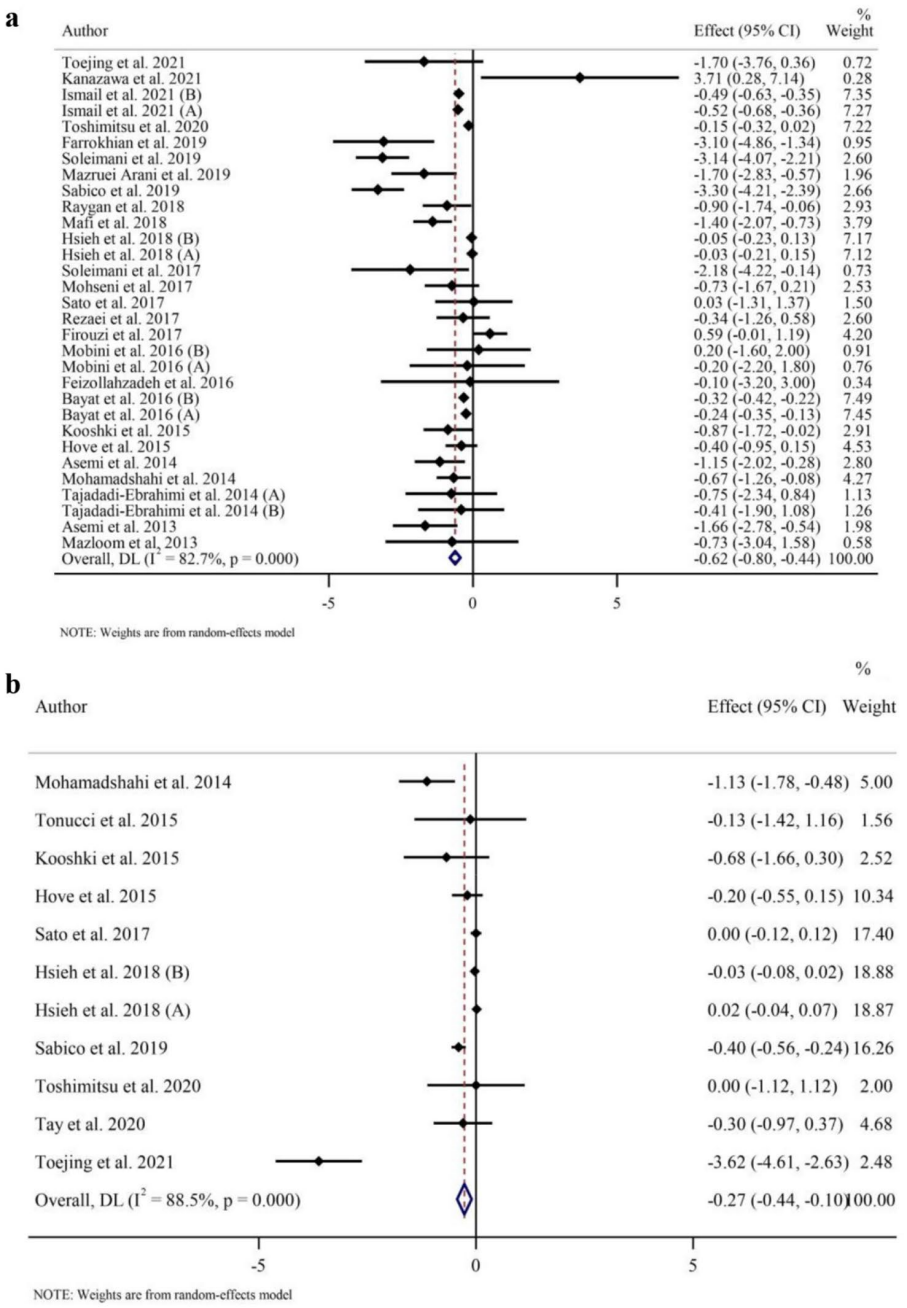

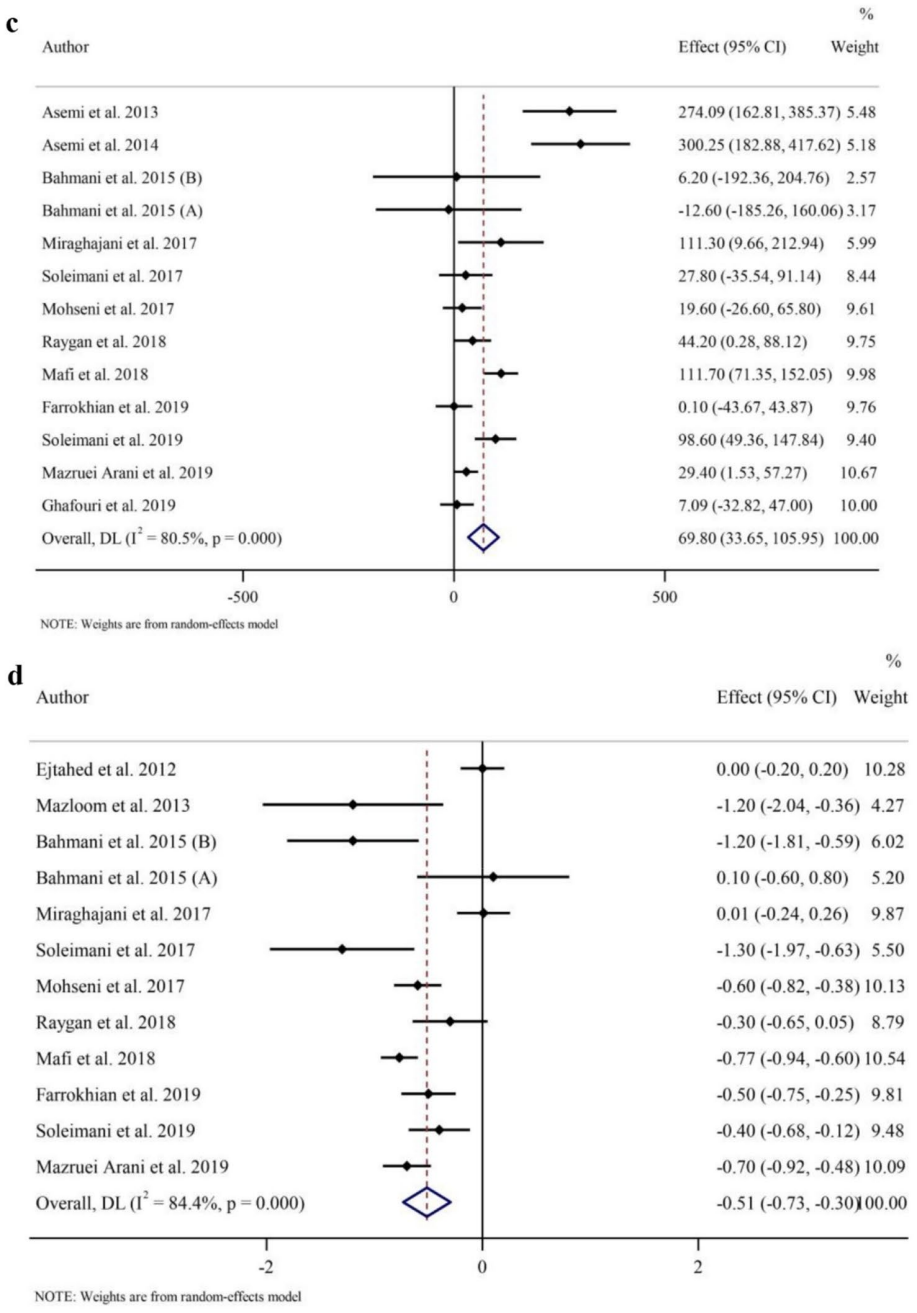

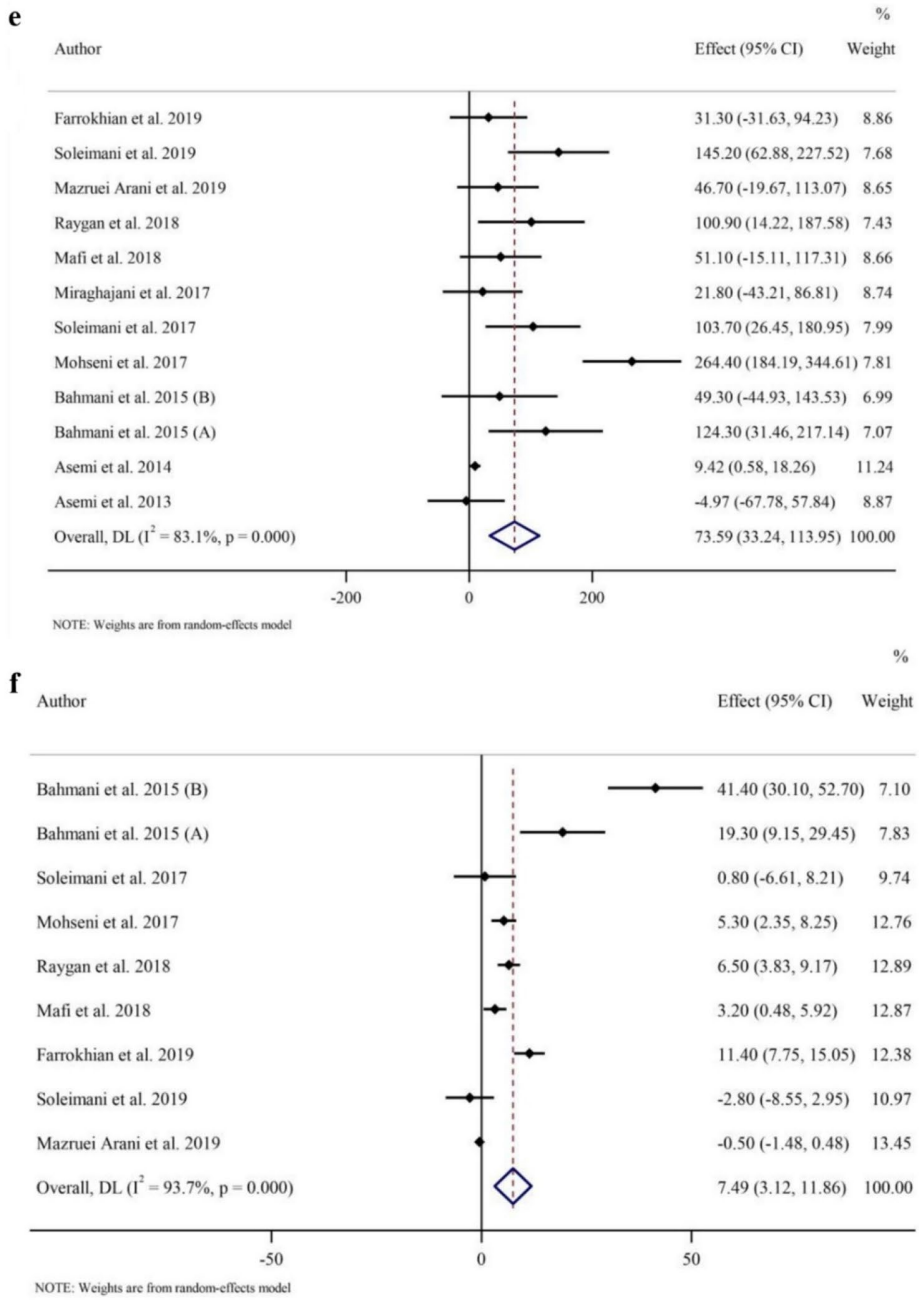
Forest plot of a random-effects meta-analysis of the effect of probiotic and synbiotic supplementation on **a** CRP; **b** TNF-α; **c** GSH **d** MDA **e** TAC **f** NO in individuals with T2DM. *CI* confidence interval, *WMD* weighted mean differences, *CRP* c-reactive Protein, *TNF-α* tumor necrosis factor-α, *GSH* glutathione, *MDA* malondialdehyde, *TAC* total antioxidant capacity, *NO* nitric oxide, *T2DM* type 2 diabetes mellitus

Twenty-six RCTs with 31 effect sizes evaluated CRPs as an outcome measure (intervention samples = 945/control samples = 824). Probiotic and synbiotic supplementation resulted in a reduction in CRPs (WMD − 0.62 mg/l; 95% CI − 0.80, − 0.44; *p* < 0.001) compared to placebo group and a large between-study heterogeneity was observed (*I*^2^ = 82.7%, *p* < 0.001) (Fig. 2a). According to the subgroup analyses, probiotic and synbiotic supplementation significantly decreased CRP in all subgroups except in studies among patients with normal baseline BMI (18.5–24.9 kg/m^2^) and individuals with prediabetes (Table 3), showing more potent effects in patients with T2DM (*p* < 0.001) compared to those with prediabetes (*p* = 0.07) and individuals with overweight (*p* < 0.001) and obesity (*p* = 0.001).

**Table 3:** Subgroup analyses of probiotics and synbiotic supplementation on inflammatory cytokines, oxidative stress and adipokines

	Number of studies	WMD (95%CI)	*p* value	heterogeneity		
				*p* heterogeneity	*I*^2^	*p* between
*Subgroup analyses of probiotic and synbiotic supplementation on CRP*						
Overall effect	31	− 0.62 (− 0.80, − 0.44)	< 0.001	< 0.001	82.7%	
Baseline CRP (mg/l)						
< 3	9	− 0.26 (− 0.43, − 0.09)	0.003	0.007	62.3%	0.001
≥ 3	18	− 1.22 (− 1.77, − 0.68)	< 0.001	< 0.001	74.0%	
Trial duration (week)						
< 12	11	− 038 (− 053, − 0.23)	< 0.001	0.167	29.3%	0.031
≥ 12	20	− 0.73 (− 1.01, − 0.45)	< 0.001	< 0.001	88.0%	
Health status						
T2DM	30	− 0.67 (− 0.86, − 0.47)	< 0.001	< 0.001	82%	< 0.001
Pre T2DM	1	− 0.15 (− 0.32, 0.02)	0.076	−	0.0%	
Supplementation						
Probiotic	25	− 0.47 (− 0.64, − 0.30)	< 0.001	< 0.001	79.6%	0.202
Synbiotic	6	− 1.27 (− 2.49, − 0.05)	0.041	< 0.001	82.7%	
Baseline BMI (kg/m^2^)						
Normal (18.5–24.9)	4	− 0.39 (− 0.91, 0.14)	0.146	0.182	38.3%	0.285
Overweight (25–29.9)	17	− 0.76 (− 1.03, − 0.48)	< 0.001	< 0.001	86.1%	
Obese (> 30)	8	− 1.00 (− 1.58, − 0.43)	0.001	0.016	59.4%	
*Subgroup analyses of probiotic and synbiotic supplementation on TNF-α*						
Overall effect	12	− 0.48 (− 0.81, − 0.15)	0.004	< 0.001	84.8%	
Trial duration (week)						
< 12	3	− 0.86 (− 1.36, − 0.35)	0.001	0.362	1.6%	0.156
≥ 12	9	− 0.41 (− 0.77, − 0.05)	0.023	< 0.001	87.3%	
Health status						
T2DM	9	− 0.55 (− 0.91, − 0.18)	0.002	< 0.001	88.9%	0.345
Pre T2DM	3	− 0.22 (− 0.79, 0.35)	0.455	0.904	0.0%	
Supplementation						
Probiotic	11	− 0.47 (− 0.81, − 0.13)	0.006	< 0.001	86.0%	0.697
Synbiotic	1	− 0.68 (− 1.66, 0.30)	0.176	–	0.0%	
Baseline BMI (kg/m^2^)						
Normal (18.5–24.9)	5	− 0.88 (− 2.29, 0.52)	0.217	< 0.001	92.3%	0.758
Overweight (25–29.9)	4	− 0.44 (− 0.75, − 0.12)	0.006	0.098	52.4%	
Obese (> 30)	1	− 0.30 (− 0.97, 0.37)	0.383	–	–	
*Subgroup analyses of probiotic and synbiotic supplementation on IL-6*						
Overall effect	13	− 0.12 (− 0.40, 0.16)	0.391	0.014	52.3%	
Trial duration (week)						
< 12	5	0.12 (− 0.61, 0.85)	0.749	0.025	64%	0.375
≥ 12	8	− 0.24 (− 0.57, 0.08)	0.146	0.113	39.8%	
Health status						
T2DM	11	− 0.05 (− 0.46, 0.36)	0.814	0.020	52.9%	0.482
Pre T2DM	2	− 0.27 (− 0.76, 21.86)	0.258	0.088	65.7%	
Supplementation						
Probiotic	11	− 0.04 (− 0.34, 0.25)	0.772	0.019	53.2%	0.085
Synbiotic	2	− 0.58 (− 1.12, − 0.04)	0.033	0.647	0.0%	
Baseline BMI (kg/m^2^)						
Normal (18.5–24.9)	4	− 0.20 (− 0.76, 0.34)	0.461	0.113	49.7%	0.854
Overweight (25–29.9)	5	− 0.20 (− 0.67, 0.26)	0.396	0.102	48.3%	
Obese (> 30)	2	0.40 (− 1.70, 2.52)	0.705	0.002	89.9%	
*Subgroup analyses of probiotic and synbiotic supplementation on Adiponectin*						
Overall effect	6	0.66 (− 0.44, 1.77)	0.240	0.031	59.4%	
*Subgroup analyses of probiotic and synbiotic supplementation on leptin*						
Overall effect	5	− 2.29 (− 5.73, 1.15)	0.192	< 0.001	82.0%	
*Subgroup analyses of probiotic and synbiotic supplementation on GSH*						
Overall effect	13	69.80 (33.65, 105.95)	< 0.001	< 0.001	80.5%	
Trial duration (week)						
< 12	6	119.58 (0.39, 238.76)	0.049	< 0.001	87.2%	0.272
≥ 12	7	47.51 (16.50, 78.52)	0.003	0.001	72.4%	
Supplementation						
Probiotic	8	68.72 (26.84, 110.61)	0.001	< 0.001	77.2%	0.752
Synbiotic	5	85.14 (− 7.64, 177.92)	0.056	< 0.001	91.7%	
Baseline BMI (kg/m^2^)						
Overweight (25–29.9)	8	77.53 (27.47, 127.58)	0.002	< 0.001	81.1%	0.532
Obese	5	58.64 (1.43, 115.84)	0.045	< 0.001	80.6%	
*Subgroup analyses of probiotic and synbiotic supplementation on MDA*						
Overall effect	12	− 0.51 (− 0.73, − 0.30)	< 0.001	< 0.001	84.4%	
Trial duration (week)						
< 12	5	− 0.36 (− 0.77, 0.06)	0.095	< 0.001	81.1%	0.272
≥ 12	7	− 0.60 (− 0.76, − 0.45)	< 0.001	0.031	56.8%	
Supplementation						
Probiotic	9	− 0.48 (− 0.76, − 0.20)	0.001	< 0.001	87.6%	0.606
Synbiotic	3	− 0.59 (− 0.93, − 0.26)	< 0.001	0.064	63.6%	
Baseline BMI (kg/m^2^)						
Overweight (25–29.9)	8	− 0.46 (− 0.77, − 0.16)	0.003	< 0.001	88.1%	0.487
Obese	4	− 0.61 (− 0.87, − 0.35)	< 0.001	0.047	62.3%	
*Subgroup analyses of probiotic and synbiotic supplementation on TAC*						
Overall effect	12	73.59 (33.24, 113.95)	< 0.001	< 0.001	83.1%	
Trial duration (week)						
< 12	5	23.64 (− 8.22, 55.50)	0.146	0.145	41.4%	0.104
≥ 12	7	103.56 (45.28, 161.84)	< 0.001	< 0.001	77.2%	
Supplementation						
Probiotic	8	85.43 (28.82, 142.04)	0.003	< 0.001	79.0%	0.791
Synbiotic	4	49.76 (− 5.81, 105.33)	0.079	0.010	73.5%	
Baseline BMI (kg/m^2^)						
Overweight (25–29.9)	7	98.32 (31.00, 165.65)	0.004	< 0.001	89.9%	0.043
Obese	5	37.02 (5.05, 68.98)	0.023	0.408	0.0%	
*Subgroup analyses of probiotic and synbiotic supplementation on NO*						
Overall effect	9	7.49 (3.12, 11.86)	0.001	< 0.001	93.7%	
Trial duration (week)						
< 12	2	30.20 (8.55, 51.86)	0.006	0.004	87.7%	0.018
≥ 12	7	3.66 (0.06, 7.25)	0.046	< 0.001	91.1%	
Supplementation						
Probiotic	6	4.52 (0.79, 8.25)	0.018	< 0.001	89.6%	0.216
Synbiotic	3	15.82 (− 1.68, 33.31)	0.076	< 0.001	95.9%	
Baseline BMI (kg/m^2^)						
Overweight (25–29.9)	5	3.99 (− 0.24, 8.22)	0.064	< 0.001	74.7%	0.085
Obese	4	12.40 (3.82, 20.98)	0.005	< 0.001	97.1%	

*CI* confidence interval, *WMD* weighted mean differences, *BMI* body mss index, *CRP* c-reactive Protein, *TNF-α* tumor necrosis factor-α, *IL-6* interleukin-6, *GSH* glutathione, *MDA* malondialdehyde, *TAC* total antioxidant capacity, *NO* nitric oxide, *T2DM* type 2 diabetes mellitus

Pooled effect sizes from 10 RCTs with 12 effect sizes (intervention samples = 299/control samples = 264) showed that TNF-α concentrations reduced (WMD − 0.48 pg/ml; 95% CI − 0.81, − 0.15; *p* = 0.004) following probiotic and synbiotic supplementation compared to placebo consumption (Fig. 2b) with a considerable between-study heterogeneity (*I*^2^ = 84.8%, *p* < 0.001). In subgroup analyses, TNF-α reduction was associated with probiotic and synbiotic supplementation regardless of the length of the trial. TNF-α levels were only reduced in individuals with T2DM (*p* = 0.002), individuals with overweight (BMI = 25–29.9 kg/m^2^) (*p* = 0.006), and when probiotic was supplemented (*p* = 0.006) (Table 3).

Based on combining 13 effect sizes including 367 intervention samples and 328 control samples, a significant heterogeneity was seen for serum IL-6 levels (*I*^2^ = 52.3%, *p* = 0.014). However, we observed that probiotic and synbiotic supplementation did not significantly affect IL-6 levels (WMD = − 0.12 pg/ml; 95% CI − 0.40, 0.16, *p* = 0.391) (Supplementary Fig. 1a). Subgroup analyses revealed that IL-6 levels were significantly reduced in synbiotic supplementation (*p* = 0.03), but not in probiotic intake (*p* = 0.7). There was no difference in IL-6 based on the health status of individuals, study duration, and baseline BMI (Table 3).

### Effects of probiotic and synbiotic intake on serum adipokines

Five and four RCTs with six and five effect sizes were investigated adiponectin (intervention samples = 137/control samples = 121) and leptin (intervention samples = 93/control samples = 74), respectively. There was no effect of probiotic and synbiotic supplementation on serum adiponectin (WMD = 0.66; 95% CI − 0.44, 1.77, *p* = 0.240) (Supplementary Fig. 1b) and leptin levels (WMD = − 2.29; 95% CI − 5.73, 1.15, *p* = 0.192) (Supplementary Fig. 1c). Significant heterogeneity between studies for both adiponectin (*I*^2^ = 59.4%, *p* = 0.031) and leptin (*I*^2^ = 82.0%, *p* < 0.001) was observed. However, subgroup analysis for adiponectin and leptin for baseline values, length of follow-up, health status of participants, type of supplementation, and baseline BMI for all subgroups was not possible due to a low number of studies (Table 3).

### Effects of probiotic and synbiotic intake on oxidative stress

As indicated in Fig. 2c, pooled data from 12 RCTs with 13 effect sizes (intervention samples = 398/control samples = 371) showed that GSH concentrations were increased with probiotic and synbiotic supplementation compared to placebo (WMD 69.80 µmol/l; 95% CI 33.65, 105.95, *p* < 0.001), with a considerable between-study heterogeneity (*I*^2^ = 80.5%, *p* < 0.001). Data from subgroup analyses showed that probiotic/synbiotic administration were associated with increased GSH irrespective of the trial duration and baseline BMI values. In addition, GSH was only reduced with supplementation with probiotic and with any duration (Table 3).

Overall, probiotic and synbiotic supplementation decreased MDA concentrations (WMD − 0.51 µmol/l; 95% CI − 0.73, − 0.30; *p* < 0.001) (Fig. 2d), with a large heterogeneity seen between studies (*I*^2^ = 84.4%, *p* < 0.001) in a meta-analysis of 11 RCTs with 12 effect sizes (intervention samples = 330/control samples = 305). The findings from the subgroup analyses showed that probiotic and synbiotic intake reduced MDA regardless of the type of intervention and baseline BMI values, but only in participants who consumed probiotic and synbiotic for twelve or more weeks probiotic and synbiotic compared to controls (Table 3).

Meta-analysis of TAC combined data from 11 studies with 12 effect sizes (intervention samples = 342/control samples = 315). Overall, probiotic and synbiotic intake increased in TAC (WMD 73.59 mmol/l; 95% CI 33.24, 113.95, *p* < 0.001) (Fig. 2e). In subgroup analyses, elevated amount of TAC following the probiotic/synbiotic supplementation was irrespective of the baseline value for BMI. Additionally, the effects were stronger with longer duration (≥ 12 weeks (*p* < 0.001)) and with probiotics (*p* = 0.003) (Table 3).

The overall findings from 8 trials with 9 effect sizes (intervention samples = 264/control samples = 237) revealed that intervention with probiotic or synbiotic significantly increased NO levels (WMD 7.49 µmol/l; 95% CI 3.12, 11.86; *p* = 0.001) (Fig. 2f) with a significant between-study heterogeneity (*I*^2^ = 93.7%, *p* < 0.001). In subgroup analyses, NO levels were elevated regardless of the length of trial but only increased in those probiotic supplementation and individuals with obesity (Table 3).

### Publication bias

We evaluated Egger’s regression test and found that there was a significant publication bias for CRP (*p* = 0.025), TNF-α (*p* = 0.034), NO (*p* = 0.024), and TAC (*p* = 0.009). However, no evidence of publication bias was observed for reports evaluating the influences of probiotic or synbiotic supplementation on IL-6 (*p* = 0.653), leptin (*p* = 0.369), adiponectin (*p* = 0.281), GSH (*p* = 0.141), and MDA (*p* = 0.619). Furthermore, there was no publication bias for CRP (*p* = 0.973), IL-6 (*p* = 0.640), TNF-α (*p* = 0.350), leptin (*p* = 0.221), adiponectin (*p* = 0.707), GSH (*p* = 0.127), NO (*p* = 0.251) and MDA (*p* = 0.945) according to Begg’s test. Publication bias was confirmed only for TAC (*p* = 0.016) based on Begg’s test. The funnel plots also proved these findings (Supplementary Fig. 2).

### Meta-regression analysis

The analysis was carried out to assess the correlation among intervention duration (weeks) of probiotic or synbiotic supplementation and CRP, IL-6, TNF-α, TAC, GSH, NO, MDA, leptin and adiponectin levels. Based on the analysis, the associations between absolute changes in these factors and the duration of the intervention were not linear (Supplementary Fig. 3).

### Grading of evidence

An evaluation of the quality of evidence using the GRADE approach is presented in Table 4. Low quality of evidence was detected for CRP, TNF-α, GSH, MDA, and NO for a very serious inconsistency (*I*^2^ = 82.7%, *I*^2^ = 88.5%, *I*^2^ = 80.5%, *I*^2^ = 84.4%, and *I*^2^ = 93.7% for heterogeneity, respectively), whereas the low quality of evidence for IL-6 and adiponectin was due to serious inconsistency (*I*^2^ = 52.3% and *I*^2^ = 59.4% for heterogeneity, respectively) and serious imprecision (wide CI). However, the evidence relating to leptin and TAC was downgraded to very low quality, because of the very serious inconsistency (*I*^2^ = 82.0% and *I*^2^ = 83.1% for heterogeneity, respectively) and serious imprecision (wide CI) for leptin and serious publication bias (*p* = 0.016) for TAC.

**Table 4:** GRADE profile of probiotics and synbiotic supplementation for on inflammatory cytokines, oxidative stress and adipokines

Outcomes	Risk of bias	Inconsistency	Indirectness	Imprecision	Publication bias	Number of intervention/control	Quality of evidence
CRP	No serious limitation	Very serious limitation^a^	No serious limitation	No serious limitation	No serious limitation	1769 (945/824)	⊕ ⊕ ◯◯ Low
TNF-α	No serious limitation	Very serious limitation^a^	No serious limitation	No serious limitation	No serious limitation	563 (299/264)	⊕ ⊕ ◯◯ Low
IL-6	No serious limitation	Serious limitation^b^	No serious limitation	Serious limitation^c^	No serious limitation	695 (367/328)	⊕ ⊕ ◯◯ Low
Adiponectin	No serious limitation	Serious limitation^b^	No serious limitation	Serious limitation^c^	No serious limitation	258 (137/121)	⊕ ⊕ ◯◯ Low
Leptin	No serious limitation	Very serious limitation^a^	No serious limitation	Serious limitation^c^	No serious limitation	167 (93/74)	⊕ ◯◯◯ Very Low
GSH	No serious limitation	Very serious limitation^a^	No serious limitation	No serious limitation	No serious limitation	779 (398/371)	⊕ ⊕ ◯◯ Low
MDA	No serious limitation	Very serious limitation^a^	No serious limitation	No serious limitation	No serious limitation	635 (330/305)	⊕ ⊕ ◯◯ Low
TAC	No serious limitation	Very serious limitation^a^	No serious limitation	No serious limitation	serious limitation^d^	657 (342/315)	⊕ ◯◯◯ Very Low
NO	No serious limitation	Very serious limitation^a^	No serious limitation	No serious limitation	No serious limitation	501 (264/237)	⊕ ⊕ ◯◯ Low

^a^There is high heterogeneity for CRP (*I*^2^ = 82.7%), TNF-α (*I*^2^ = 88.5%), leptin (*I*^2^ = 82.0%), GSH (*I*^2^ = 80.5%), MDA (*I*^2^ = 84.4%), TAC (*I*^2^ = 83.1%), and NO (*I*^2^ = 93.7%)

^b^There is moderate heterogeneity for adiponectin (*I*^2^ = 59.4%) and IL-6 (*I*^2^ = 52.3%)

^c^There is no evidence of significant effects of probiotics and synbiotic supplementation on leptin, adiponectin and IL-6 (95%CI including 0)

^d^There is evidence of publication bias for TAC (*p* = 0.016)

### Sensitivity analysis

This analysis for CRP, TNF-α, IL-6, adiponectin, leptin, GSH, MDA, TAC, and NO did not indicate evidence of sensitivity.

## Discussion

In the present meta-analysis, we evaluated the effectiveness of probiotics or synbiotics on inflammatory and oxidative stress biomarkers in patients with prediabetes and T2DM. We have demonstrated that the consumption of probiotics or synbiotics is associated with reductions in inflammatory status as measured by decreased levels of CRP, and TNF-α, without any significant changes in IL-6. Regarding oxidative stress, there was a significant decrease in MDA and an increase in TAC, GSH, and NO levels. However, probiotic or synbiotic administration did not alter leptin or adiponectin levels in individuals with prediabetes and T2DM. This suggests that the supplementation of probiotics or synbiotics could be a useful intervention to improve cardiometabolic outcomes in patients with prediabetes and T2DM.

There were five recent meta-analyses on the effects of probiotic or synbiotic supplementation on inflammatory and oxidative stress biomarkers. These focused mainly on patients with diabetic nephropathy [16, 56] or investigated the effects of only probiotic supplementation [16–18, 57, 58]. In addition, two meta-analyses investigated the effectiveness of probiotics or synbiotics supplementation on various outcomes. The endpoints for the first one were CRP, TAC, MDA, NO, and GSH among individuals with T2DM [56], while the studied outcomes for the other one were TNF-α, CRP, IL-6, and NO among subjects with diabetes [59]. In other words, they included fewer RCTs and limited indicators of inflammation and oxidative stress. The current meta-analysis is also the first GRADE-assessed study summarizing publications on the effects of probiotics or synbiotics supplementation on biomarkers of inflammation, oxidative stress, and circulating adipokines levels in individuals with prediabetes and T2DM. Our subgroup analysis based on study duration indicated that both short (< 12 weeks) and long-term (≥ 12 weeks) supplementation led to a significant improvement in CRP, TNF-α, GSH, and NO following the interventions. However, probiotics or synbiotics had more favorable effects on MDA and TAC when interventions were longer than 12 weeks. Moreover, the modulating effects of probiotics or synbiotics on inflammation (CRP and TNF-α levels) were more pronounced in patients with T2DM compared to individuals with prediabetes. This suggests that the effects of probiotics or synbiotics are more pronounced in patients with heightened inflammation. Probiotic or synbiotic products also showed favorable anti-oxidative effects in individuals with T2DM and prediabetes in overweight and obese populations by a significant decrease in MDA and an increase in TAC and GSH concentrations. However, a decline in NO level was more significant in patients with obesity than overweight patients or those with normal baseline BMI. Additionally, probiotics or synbiotics did not improve inflammatory status (CRP and TNF-α) in individuals with normal body weight, suggesting that probiotics or synbiotics consumption may not be beneficial in patients with normal body weight and/or BMI.

Observational studies provided further evidence on the link between inflammation and T2DM. A meta-analysis by Wang et al. showed that elevated levels of pro-inflammatory markers, including IL-6 and CRP, are significantly associated with an increased risk of T2DM [60]. Moreover, previous studies showed the positive effects of probiotics or synbiotics in improving glycemic profile and control (HBA1c) in patients with T2DM [61]. This was confirmed by a meta-analysis of 18 RCTs in patients with T2DM that showed that probiotics improved glycemic profile by reducing glucose, insulin, and HbA1c [62]. These findings suggest that probiotics or synbiotics reduce chronic low-grade inflammation associated with T2DM, which may result in a lower risk of diabetes complications [63, 64].

However, several meta-analyses have been performed on the effects of probiotics or synbiotics on biomarkers of oxidative stress. Pourrajab et al. reported that probiotic or synbiotic supplementation could significantly increase serum TAC, GSH, and NO and reduce MDA levels in adults [65]. Likewise, Hemati et al. also showed that probiotic supplementation improved antioxidant resistance and increase antioxidant enzymes in the body by increasing TAC, GSH, SOD, and NO and decreasing MDA in various populations [66]. Similar findings have been reported in other meta-analyses [67, 68].

The findings of the previous systematic reviews and meta-analyses (with smaller sample sizes) showed that probiotics or synbiotics supplementation might help improve biomarkers of oxidative stress by decreasing MDA and increasing TAC, GSH, and NO and alleviate inflammation through a decline in CRP and TNF-α with no change in IL-6 levels [56, 59]. Our findings were similar to the previous meta-analyses, which underlined the favorable effects of probiotics or synbiotics consumption on inflammatory and oxidative stress biomarkers.

The mechanisms underlying the modulation effects of probiotics or synbiotics on inflammation and oxidative stress remain largely unclear. However, we postulate four possible explanations for the relationship between probiotics or synbiotics and inflammation and oxidative stress. First, intestinal microorganisms produce short-chain fatty acids (SCFAs) [69]. The production of SCFAs can decrease the enzymatic synthesis of CRP in the liver [70]. Second, reports have shown that hyperglycemia stimulates the nuclear factor kappa B (NF-kB) pathway. The suppression of NF-kB pathway results in decreased levels of pro-inflammatory cytokine IL-6 [71]. IL-6 induces CRP gene expression and inhibits the NF-kB/IL-6 pathway which results in decreased CRP [72, 73]. Previous studies showed that probiotics or synbiotics might have hypoglycemic properties [74]; therefore, probiotic or synbiotic supplementation might modulate inflammation and oxidative stress by controlling blood glucose. Third, dyslipidemia in patients with T2DM and prediabetes is closely linked to inflammation and oxidative stress [75]. Since the lipid profile-improving influences of probiotic or synbiotic supplementation have been well documented [36, 55, 76–78], these supplements may reduce the biomarkers of inflammation and oxidative stress. Fourth, the antioxidative effects of probiotics or synbiotics have been reported [79]. It is well-known that antioxidants can modulate oxidative stress and inflammation [80]. While these findings are attention grabbing, further research is still needed to verify and define the possible mechanisms related to the effects of probiotics or synbiotics on oxidative stress and inflammation in patients with T2DM and prediabetes.

## Strengths and limitations

This meta-analysis appears to contain many strengths and some limitations. The high number of studies and high overall sample size is the main strength of this study. Moreover, we analyzed a wide range of inflammatory and oxidative stress biomarkers linked to the pathogenesis of T2DM.

There is no publication bias in the analysis. In addition, a meta-regression analysis was performed to assess the association between pooled effect sizes, doses, and supplementation periods. Finally, based on the GRADE guidelines, we graded the overall certainty of evidence across the studies. Regarding limitations, since all the trials except one were equal to or less than 3 months, our analysis cannot assess the long-term effects of probiotic or synbiotic supplementation on inflammation and oxidative stress profile and circulating adipokines level. Moreover, our analysis showed high statistical heterogeneity. This may be due to a variety of methodologies (different study designs) and/or differences in treatment regimens (doses/durations) or the intervention type (probiotic or synbiotic). In addition, the number of studies conducted on patients with prediabetes was limited. Finally, many clinical studies included in the current study were from Iran, limiting the study to reflect diverse populations worldwide and generalizing the results.

## Conclusions

In conclusion, our findings show that probiotics or synbiotics intake may reduce cardiovascular disease risk in patients with prediabetes and T2DM, by decreasing CRP, TNF-α, and MDA and increasing TAC, GSH, and NO levels, but have no significant effects on IL-6, adiponectin, and leptin when compared with a control group. Patients with T2DM seem to benefit more from this intervention than individuals with prediabetes. In addition, probiotic or synbiotic products also showed favorable anti-oxidative effects in individuals with T2DM and prediabetes in overweight and obese populations. Large-scale RCTs with longer follow-ups are necessary to establish the long-term effects of these supplements in both prediabetes and T2DM. Furthermore, investigating the mechanisms involved in probiotic or synbiotic effects on the studied outcomes is crucial to determining how these interventions target specific signaling pathways.

## Supplementary Information

Below is the link to the electronic supplementary material.

Supplementary file1 (DOCX 1940 KB)

## Data Availability

The datasets generated and analyzed during the current study are available from the corresponding author on reasonable request.
