# Supplementary material for: The effects of probiotic and synbiotic supplementation on inflammation, oxidative stress, and circulating adiponectin and leptin concentration in subjects with prediabetes and type 2 diabetes mellitus: a GRADE-assessed systematic review, meta-analysis, and meta-regression of randomized clinical trials
Source: Eur J Nutr. 2022 Oct 14;62(2):543–61. doi: 10.1007/s00394-022-03012-9 (PMC9941248; doi:10.1007/s00394-022-03012-9)
Supplement: Supplementary file 1 — Supplementary file1 (DOCX 1940 KB) [file 394_2022_3012_MOESM1_ESM.docx]

**
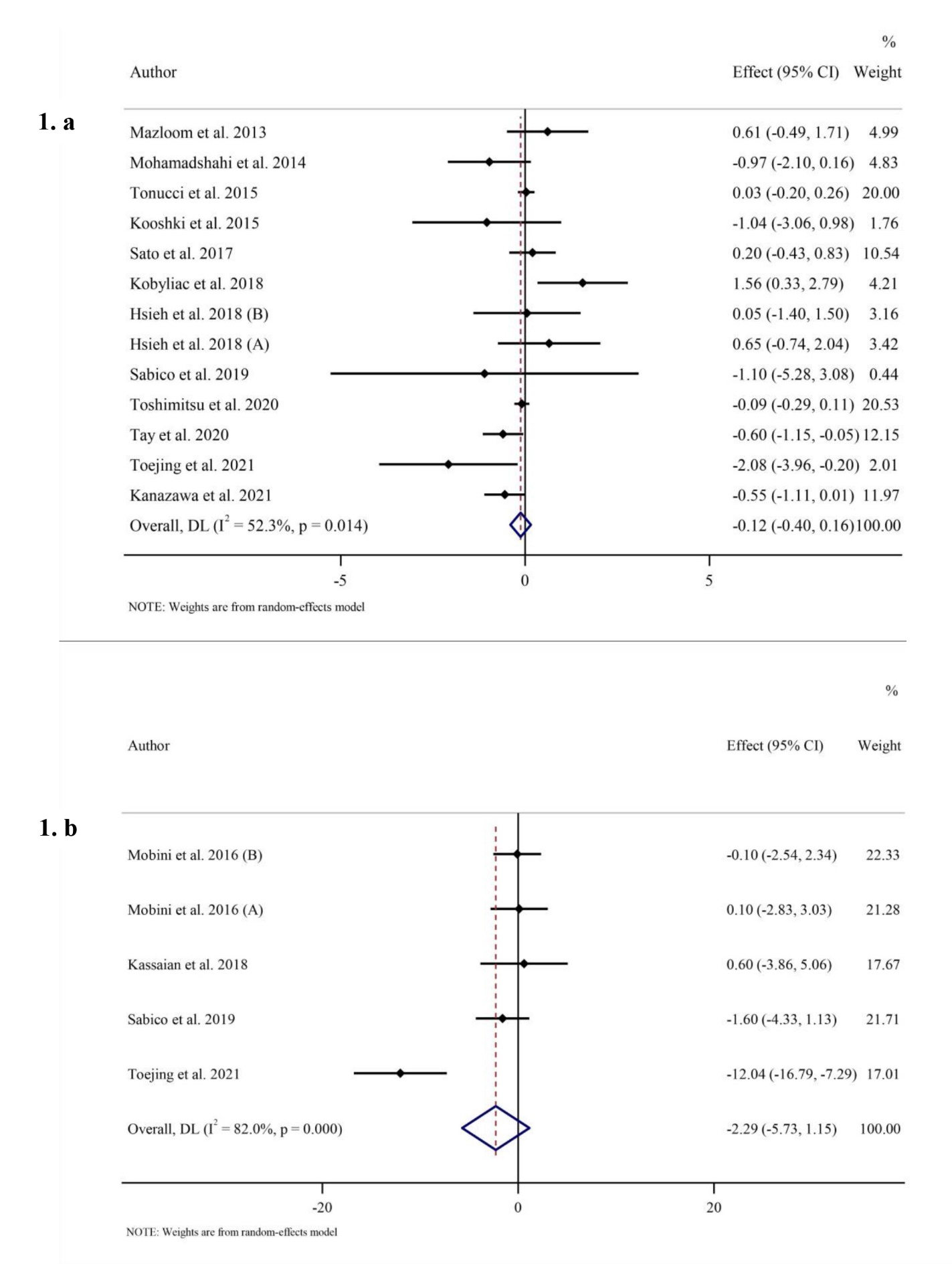
**

**
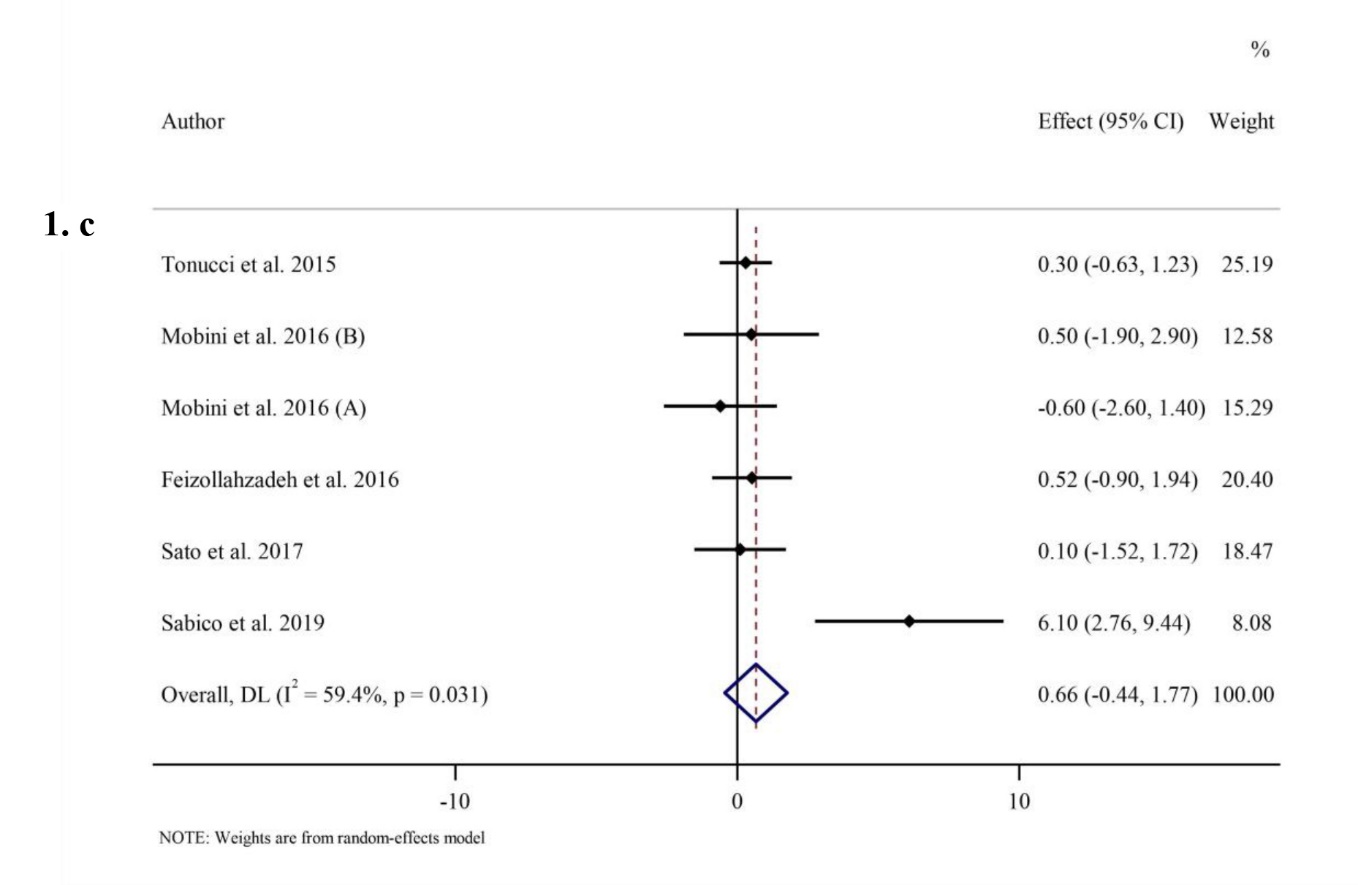
**

**Figure 1.** Forest plot of a random-effects meta-analysis of the effect of probiotic or synbiotic supplementation on (a) Interleukin-6 (IL-6); (b) leptin; (c) adiponectin; WMD, weighted mean difference.

**
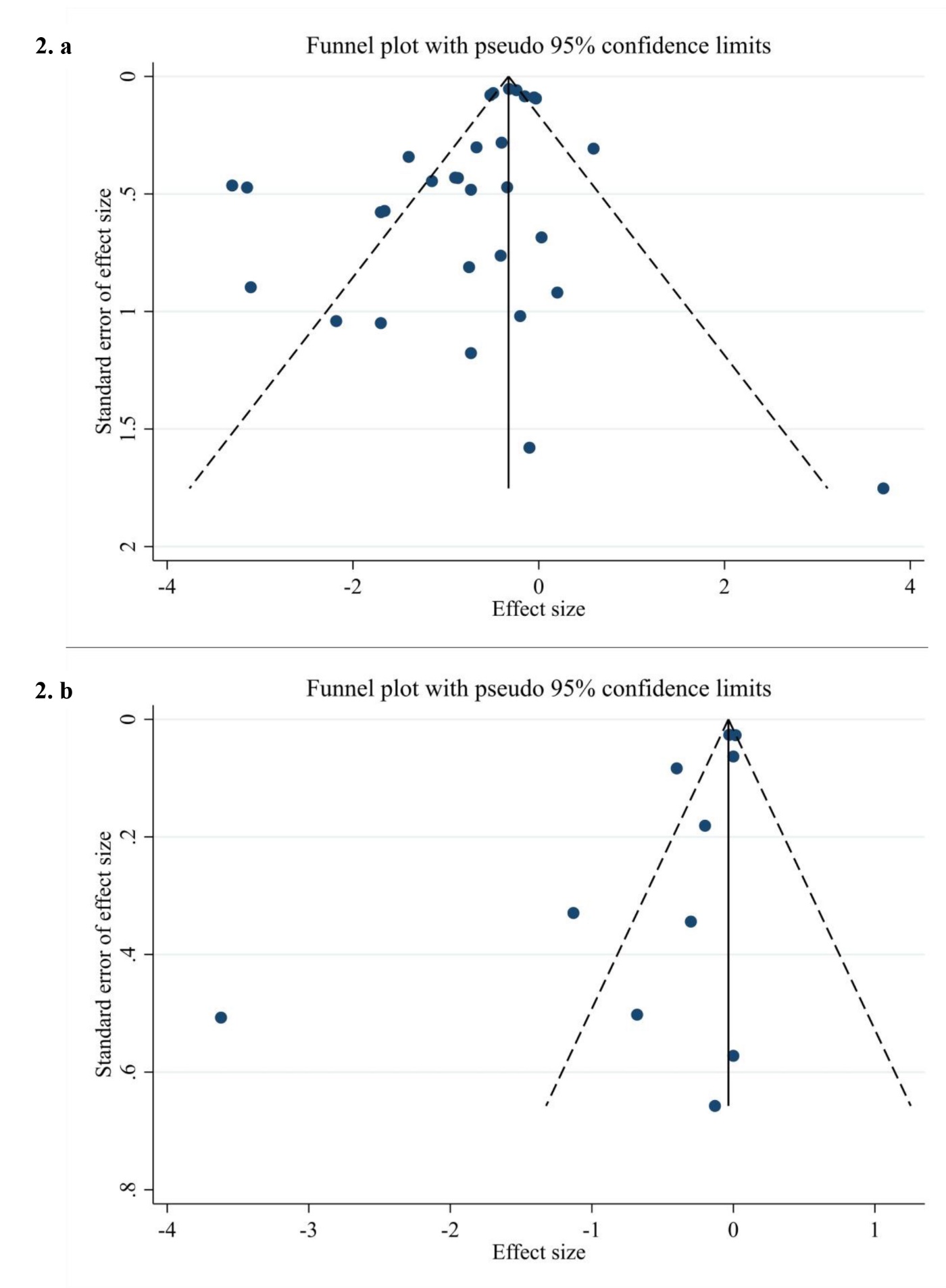
**

**
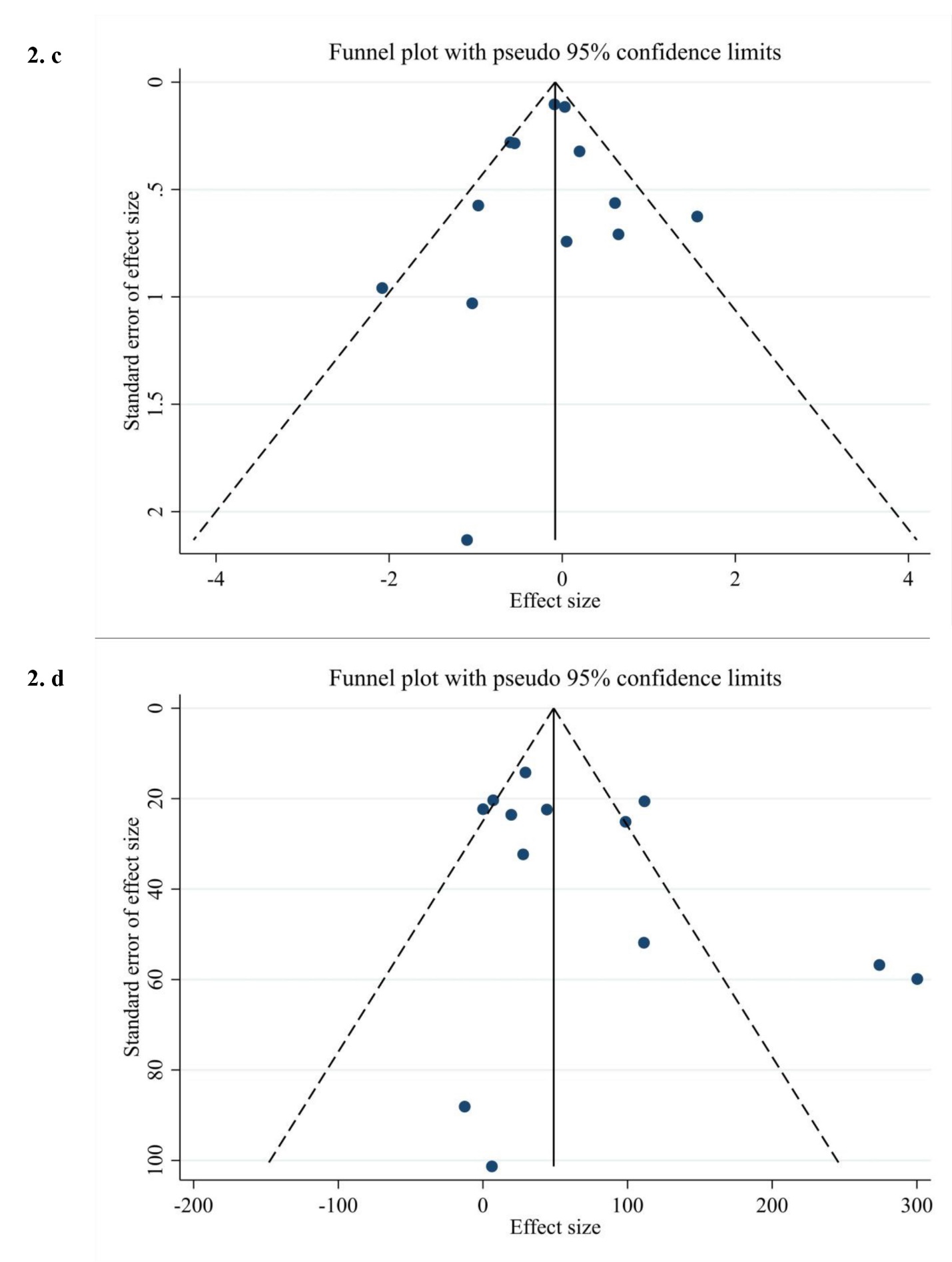
**

**
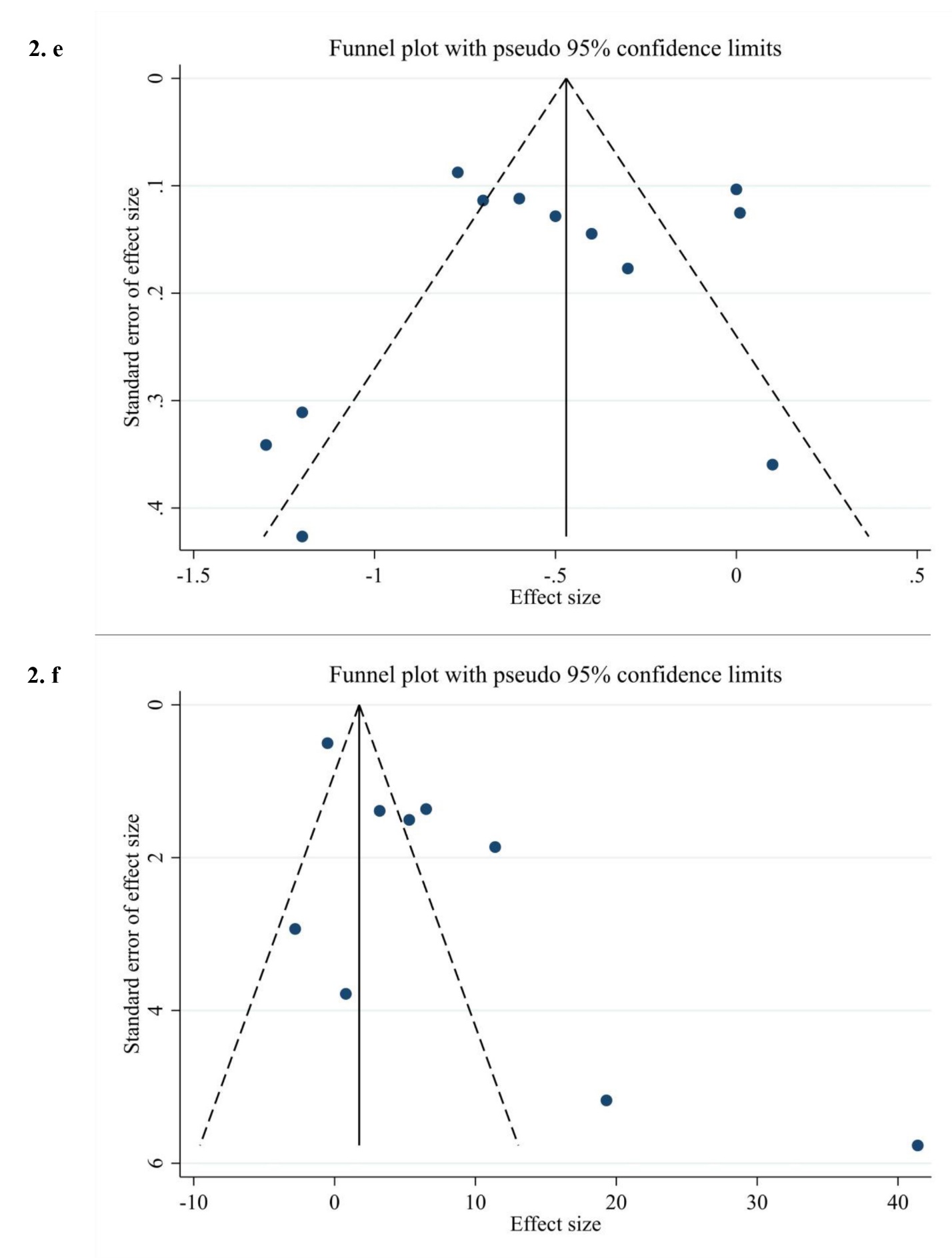
**

**
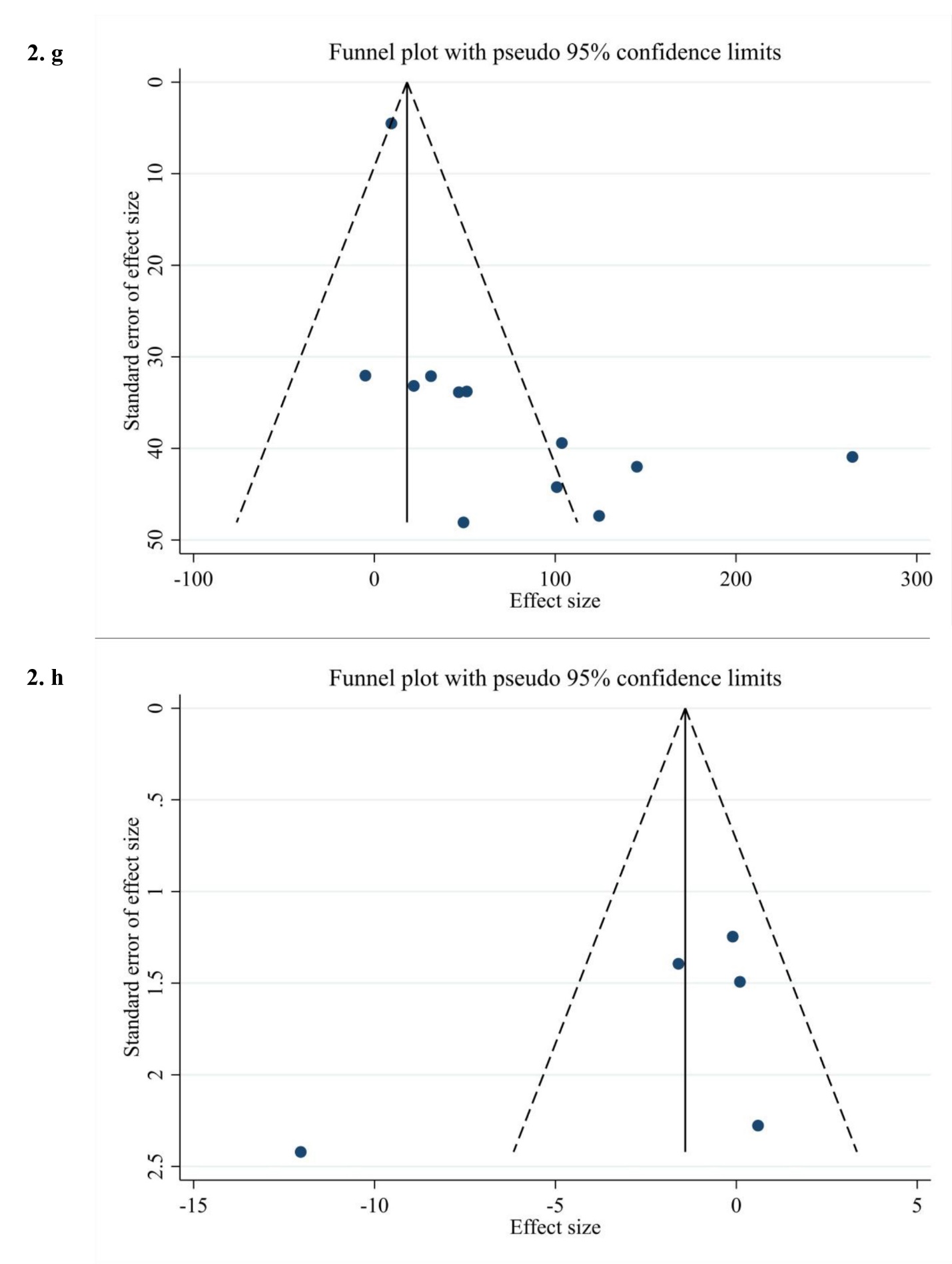
**

**
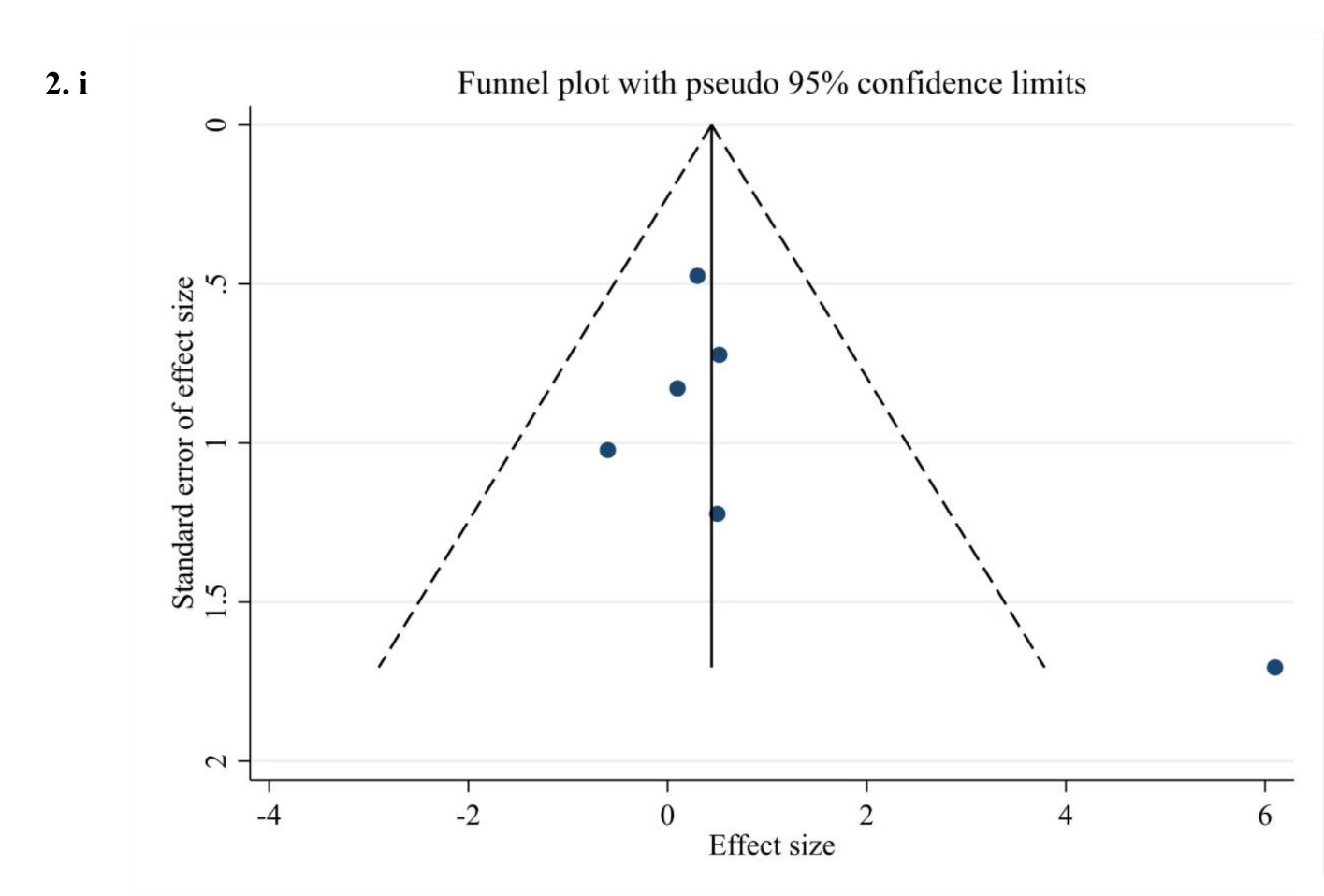
**

**Figure 2.** Funnel plot for the effect of probiotic and synbiotic supplementation on; (A) CRP; (B) TNF-α; (C) IL-6; (D) GSH (E) MDA (F) NO (G) TAC (H) leptin (I) Adiponectin. Abbreviations: CRP, c-reactive Protein; TNF-α, tumor necrosis factor-α; IL-6, interlukin-6; GSH, glutathione; MDA, malondialdehyde; NO, nitric oxide; TAC, total antioxidant capacity.


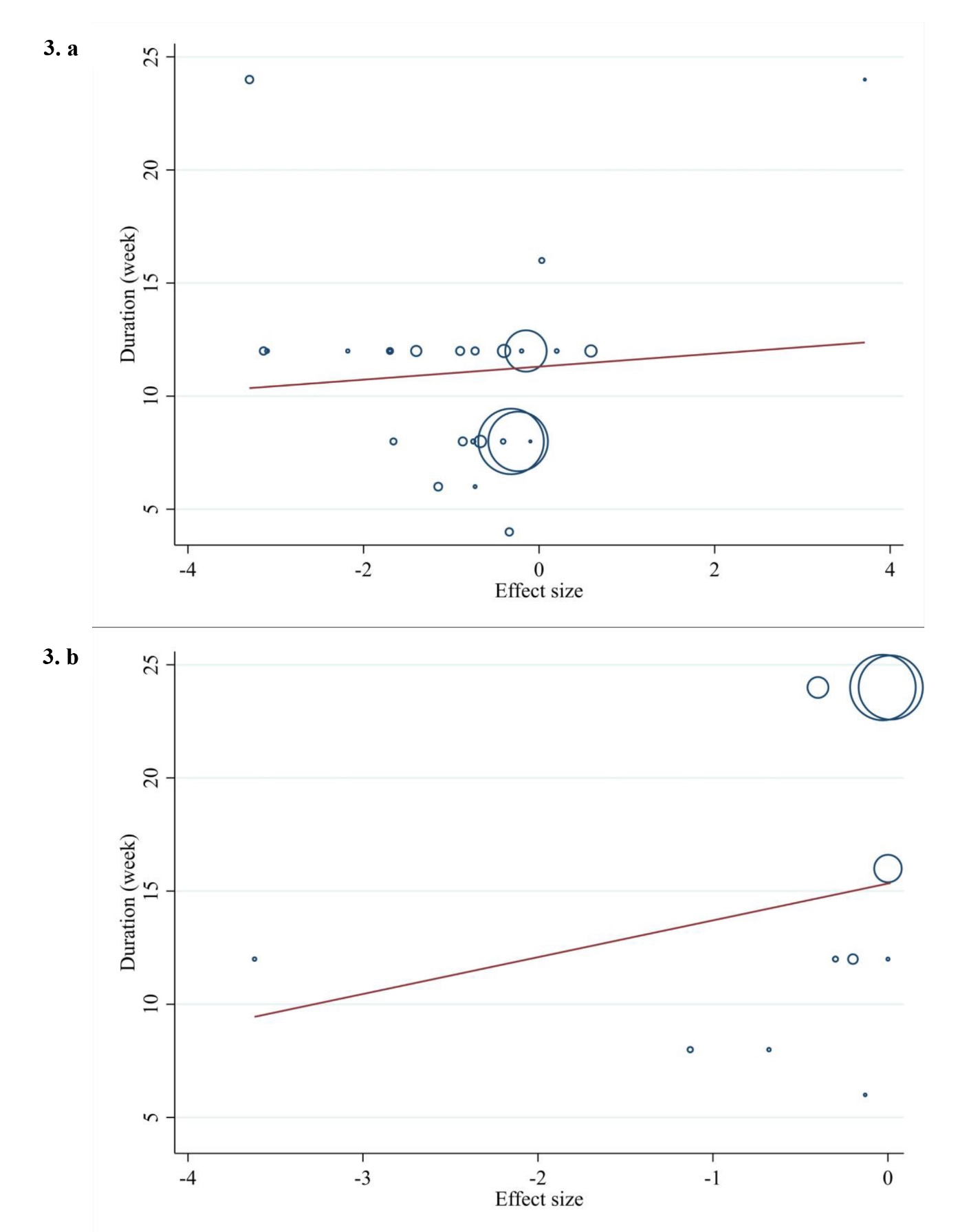


**
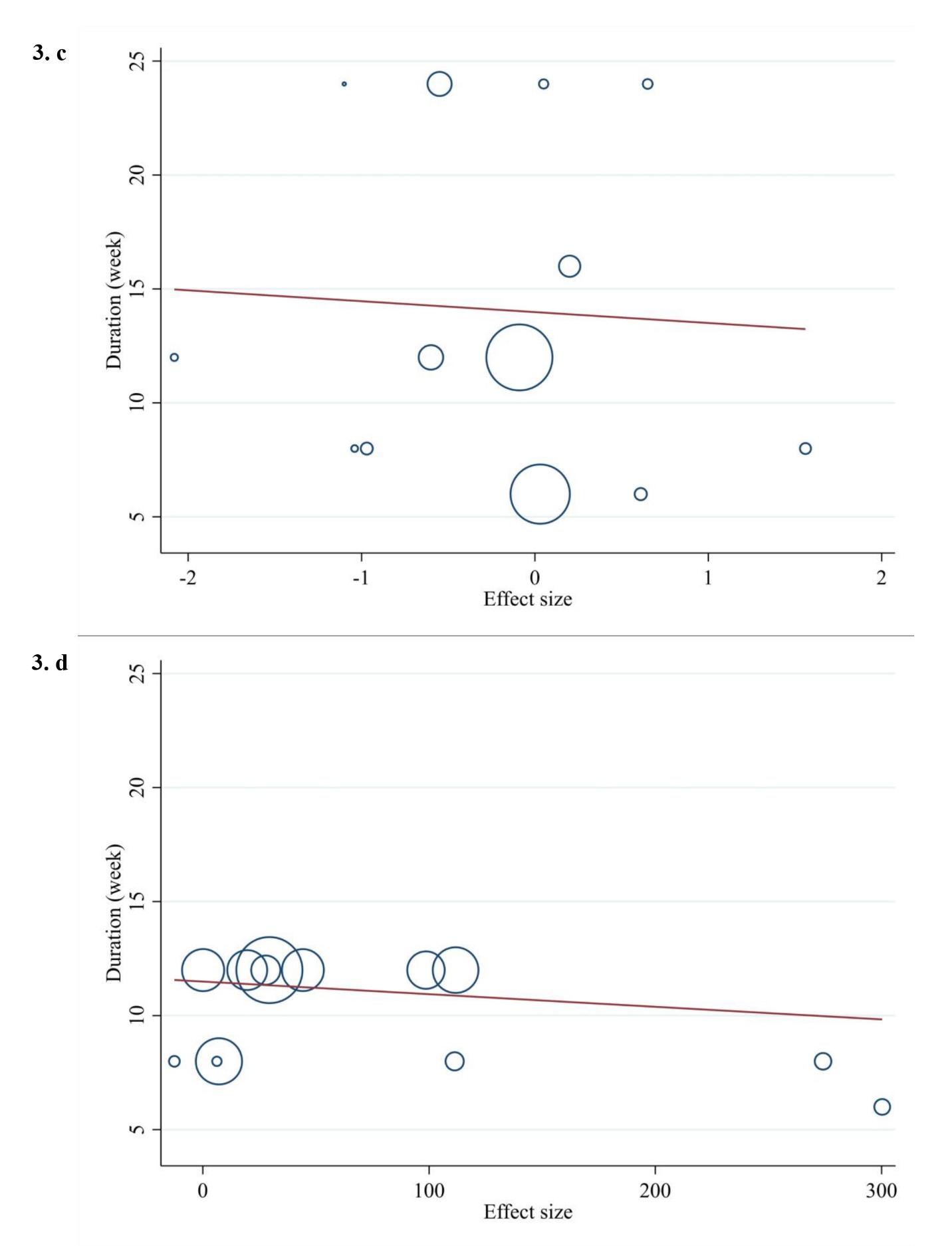
**

**
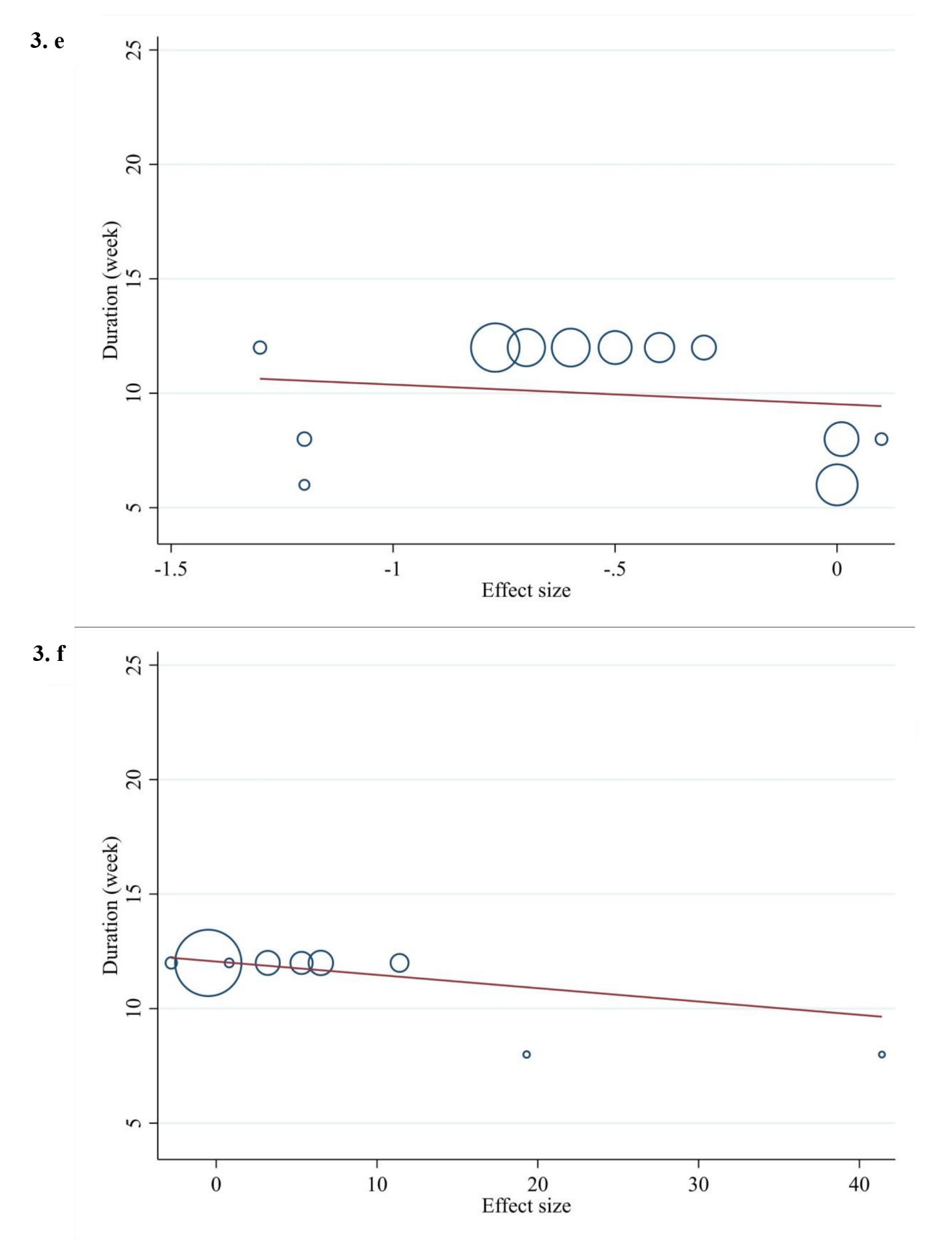
**

**
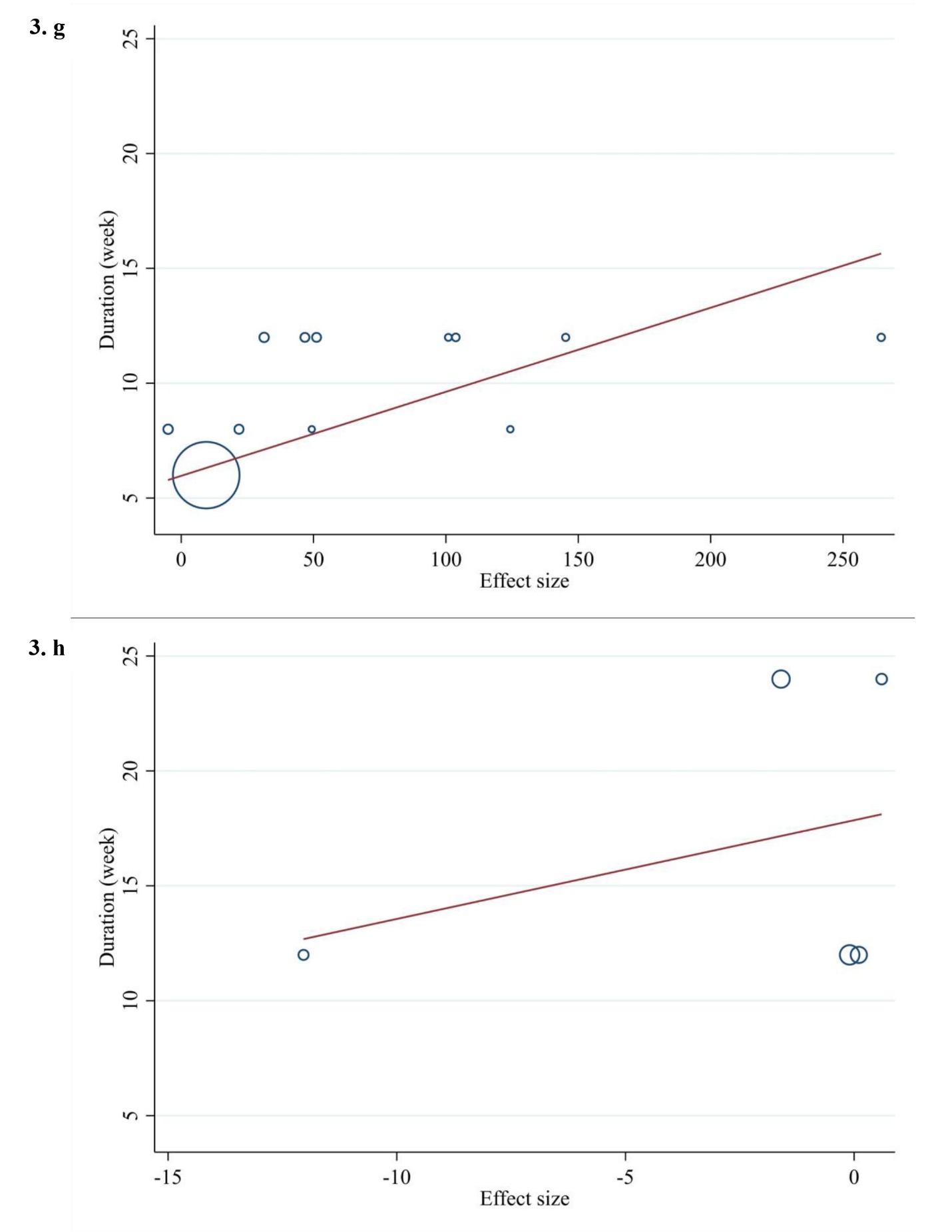
**

**
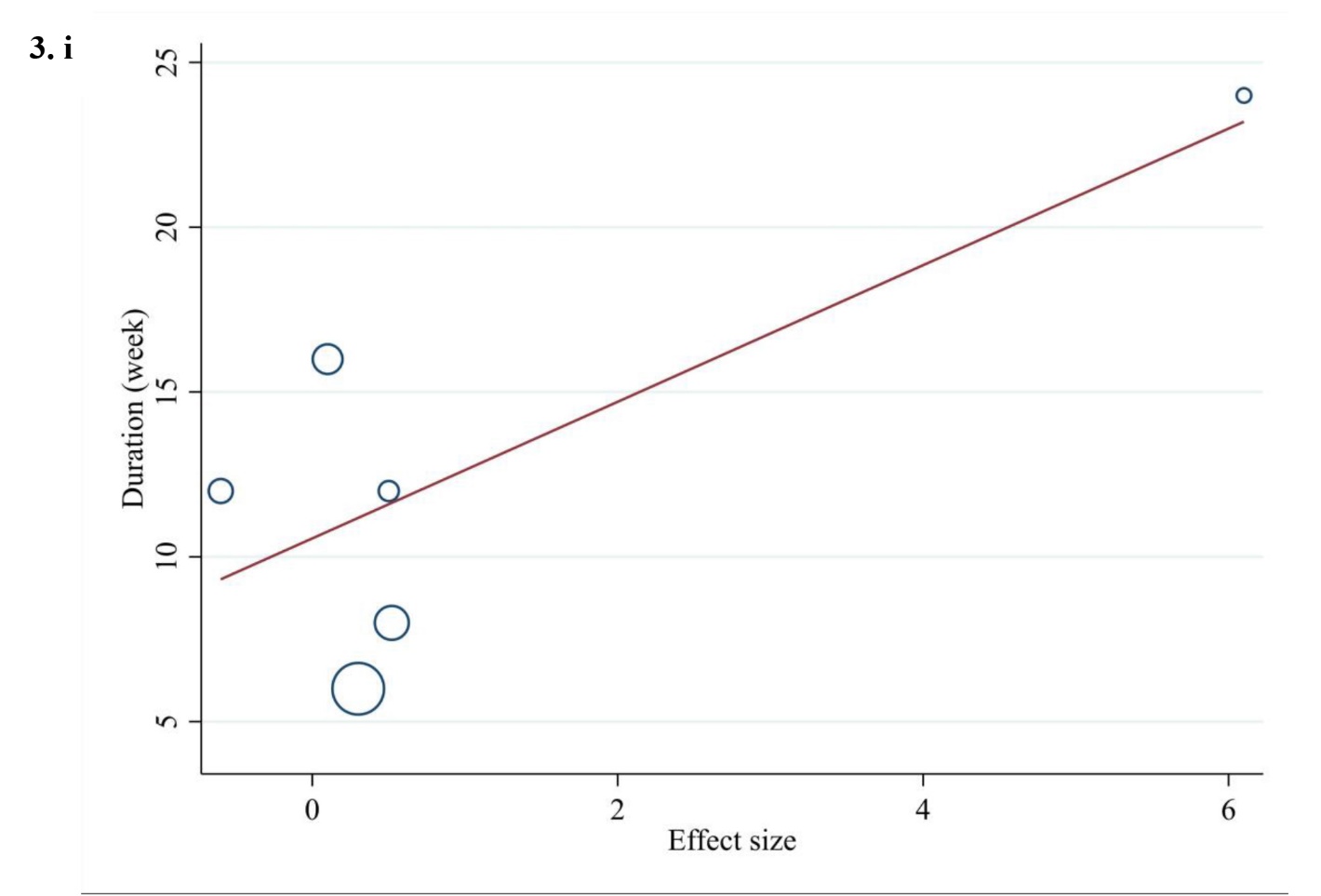
**

**Figure 3.** Random-effects meta-regression plots of the correlation between intervention duration of probiotic or synbiotic supplementation and weighted mean difference of (A) CRP; (B) TNF-α; (C) IL-6; (D) GSH (E) MDA (F) NO (G) TAC (H) leptin (I) Adiponectin. Abbreviations: CRP, c-reactive Protein; TNF-α, tumor necrosis factor-α; IL-6, interlukin-6; GSH, glutathione; MDA, malondialdehyde; NO, nitric oxide; TAC, total antioxidant capacity.

**Table 1.** Search terms used across the various databases

| PubMed | (Probiotics[Title/Abstract] OR probiotic[Title/Abstract] OR Synbiotics[Title/Abstract] OR synbiotic[Title/Abstract] OR Lactobacillus[Title/Abstract] OR Bifidobacterium[Title/Abstract]) AND (Intervention[Title/Abstract] OR "controlled trial"[Title/Abstract] OR randomized[Title/Abstract] OR random[Title/Abstract] OR randomly[Title/Abstract] OR placebo[Title/Abstract] OR "clinical trial"[Title/Abstract] OR Trial[Title/Abstract] OR "randomized clinical trial"[Title/Abstract] OR RCT[Title/Abstract] OR trial[Title/Abstract] OR trials "Cross-Over Studies"[Title/Abstract] OR "Cross-Over"[Title/Abstract] OR "Cross-Over Study"[Title/Abstract] OR parallel[Title/Abstract] OR "parallel study"[Title/Abstract] OR "parallel trial"[Title/Abstract]) |
| --- | --- |
| Scopus | ( TITLE-ABS-KEY ( probiotics OR probiotic OR synbiotics OR synbiotic OR lactobacillus OR bifidobacterium ) AND TITLE-ABS-KEY ( intervention OR "controlled trial" OR randomized OR random OR randomly OR placebo OR "clinical trial" OR trial OR "randomized clinical trial" OR rct OR trial OR trials "Cross-Over Studies" OR "Cross-Over" OR "Cross-Over Study" OR parallel OR "parallel study" OR "parallel trial" ) ) |
| Web of science | TITLE: (Probiotics OR probiotic OR Synbiotics OR synbiotic OR Lactobacillus OR Bifidobacterium) AND TITLE: ("controlled trial" OR randomized OR random OR randomly OR placebo OR "clinical trial" OR Trial OR "randomized clinical trial" OR RCT OR trial OR trials "Cross-Over Studies" OR "Cross-Over" OR "Cross-Over Study" OR parallel OR "parallel study" OR "parallel trial") |

**Table 2.** Risk of bias assessment.

| **studies** | **Random sequence generation** | **Allocation concealment** | **Selective reporting** | **Other sources of bias** | **Blinding (participants and personnel)** | **Blinding (outcome assessment)** | **Incomplete outcome data** | **General risk of bias** |
| --- | --- | --- | --- | --- | --- | --- | --- | --- |
| Toejing et al. 2021 | L | L | H | U | L | U | L | Low |
| Kanazawa et al. 2021 | L | L | H | U | H | H | L | Low |
| Ismail et al. 2021 (A) | U | U | L | U | U | U | L | Low |
| Ismail et al. 2021 (B) | U | U | L | U | U | U | L | Low |
| Tay et al. 2020 | L | L | L | H | L | U | L | Low |
| Toshimitsu et al. 2020 | L | L | H | L | L | U | L | Low |
| Farrokhian et al. 2019 | L | L | H | L | L | U | L | Low |
| Mazruei Arani et al. 2019 | L | L | H | U | L | U | L | Low |
| Soleimani et al. 2019 | L | L | L | U | L | U | L | Low |
| Sabico et al. 2019 | L | L | L | L | L | U | L | Low |
| Raygan et al. 2018 | L | L | L | L | L | U | L | Low |
| Kobyliac et al. 2018 | L | L | L | U | L | U | L | Low |
| Mafi et al. 2018 | L | L | L | U | L | U | L | Low |
| Hsieh et al. 2018 (A) | L | L | L | U | L | U | H | Low |
| Hsieh et al. 2018 (B) | L | L | L | U | L | U | H | Low |
| Firouzi et al.2017 | L | L | L | L | L | U | L | Low |
| Miraghajani et al. 2017 | L | L | L | L | H | U | L | Low |
| Soleimani et al. 2017 | L | L | L | U | L | U | L | Low |
| Rezaei et al. 2017 | L | L | H | U | L | U | L | Low |
| Mohseni et al. 2017 | L | L | L | U | L | U | L | Low |
| Sato et al. 2017 | L | L | H | U | L | U | L | Low |
| Feizollahzadeh et al. 2016 | L | L | H | L | L | U | H | Low |
| Bayat et al. 2016 (A) | L | L | L | L | U | U | H | Low |

**Table 2.** Continued.

| **studies** | **Random sequence generation** | **Allocation concealment** | **Selective reporting** | **Other sources of bias** | **Blinding (participants and personnel)** | **Blinding (outcome assessment)** | **Incomplete outcome data** | **General risk of bias** |
| --- | --- | --- | --- | --- | --- | --- | --- | --- |
| Bayat et al. 2016 (B) | L | L | L | L | U | U | H | Low |
| Mobini et al. 2016 (A) | L | L | L | U | L | U | L | Low |
| Mobini et al. 2016 (B) | L | L | L | U | L | U | L | Low |
| Bahmani et al. 2015 (A) | L | L | L | U | L | U | L | Low |
| Bahmani et al. 2015 (B) | L | L | L | U | L | U | L | Low |
| Hove et al. 2015 | L | L | L | U | L | U | L | Low |
| Kooshki et al. 2015 | L | L | H | U | L | U | L | Low |
| Tonucci et al. 2015 | L | L | L | U | L | U | H | Low |
| Mohamadshahi et al. 2014 | L | L | H | L | L | U | L | Low |
| Asemi et al.  2014 | L | L | L | L | L | U | L | Low |
| Tajadadi-Ebrahimi et al. 2014 (A) | L | L | H | U | L | U | L | Low |
| Tajadadi-Ebrahimi et al. 2014 (B) | L | L | H | U | L | U | L | Low |
| Asemi et al. 2013 | L | L | L | L | L | U | L | Low |
| Mazloom et al. 2013 | L | L | L | U | L | U | L | Low |
| Ejtahed et al. 2012 | L | L | H | L | L | U | L | Low |

*General Low Risk>2 high risk, General moderate risk=high risk, General high risk<2 high risk
